# Oxygen Defect Site Filling Strategy Induced Moderate Enrichment of Reactants for Efficient Electrocatalytic Biomass Upgrading

**DOI:** 10.1002/advs.202410725

**Published:** 2024-11-04

**Authors:** Baixue Cheng, Haoyu Zhan, Yankun Lu, Danning Xing, Xingshuai Lv, Thomas Frauenheim, Peng Zhou, Shuangyin Wang, Yuqin Zou

**Affiliations:** ^1^ State Key Laboratory of Bio‐fibers and ECo─textiles College of Materials Science and Engineering Collaborative Innovation Center of Shandong Marine Biobased Fibers and Ecological Textiles Institute of Marine Biobased Materials Qingdao University Qingdao 266071 P. R. China; ^2^ State Key Laboratory of Chemo/Bio‐Sensing and Chemometrics College of Chemistry and Chemical Engineering Advanced Catalytic Engineering Research Center of the Ministry of Education Hunan University Changsha 410082 P. R. China; ^3^ Shandong Institute of Advanced Technology Jinan 250100 P. R. China; ^4^ College of Chemistry and Chemical Engineering Ocean University of China Qingdao 266100 P. R. China; ^5^ School of Science Constructor University 28759 Bremen Germany; ^6^ Beijing Computational Science Research Center Beijing 100193 P. R. China; ^7^ Institute for Advanced Study Chengdu University Chengdu 610106 P. R. China

**Keywords:** biomass upgrading, defect filling strategy, electrocatalysis, layered double hydroxides, moderate adsorptions

## Abstract

The electrocatalytic oxidation of 5‐hydroxymethylfurfural (HMF) provides a feasible approach for the efficient utilization of biomass. Defect regulation is an effective strategy in the field of biomass upgrading to enhance the adsorption capacity of reactants and thus increase the activity. However, how to select appropriate strategies to regulate the over‐enrichment of reactants induced by excessive oxygen vacancy is still a huge challenge. In this work, the defect‐filling strategy to design and construct an element‐filled oxygen vacancy site layered double hydroxide (S─Ov─LDH) is adopted, which achieves a significant reduction in the electrolysis potential of biomass platform molecule HMF oxidation reaction and a significant increase in current density. Physical characterizations, electrochemical measurements, and theoretical calculations prove that the formation of metal─S bond in the second shell effectively regulates the electronic structure of the material, thus weakening the over‐strong adsorption of HMF and OH^−^ induced by excessive oxygen vacancy, promoting the formation of high‐valence Co^3+^ during the reaction, and forming new adsorption sites. This work discusses the catalytic enhancement mechanism of defect filling in detail, fills the gap of defect filling in the field of biomass upgrading, and provides favorable guidance for the further development of defect regulation strategies.

## Graphical abstract

S‐Ov‐LDH electrocatalyst is constructed by defect filling strategy, which exhibits excellent HMF oxidation activity. The analytical results indicate that the S element filling at the defect site can promote the formation of Co^3+^ active species, weaken the over‐adsorption of reactants induced by excessive oxygen vacancies, accelerate interfacial charge transfer, and form new adsorption sites to promote the reactant transformation.

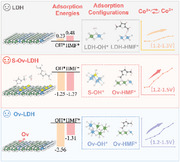

## 1 Introduction

In view of the current severe energy and environmental problems, it is imperative to develop new clean energy to balance the relationship between energy demand and environmental protection.^[^ ^1^ ^]^ As a renewable energy source with abundant resources, biomass has attracted extensive attention from researchers in recent years.^[^ ^2^ ^]^ Due to the variety of functional groups, biomass‐derived 5‐hydroxymethylfurfural (HMF) can be catalyzed into a variety of value‐added chemicals and is considered to be an important platform compound.^[^ ^3^ ^]^ Among many catalytic downstream products, 2,5 furanodicarboxylic acid (FDCA) is used as a green substitute for petroleum‐based terephthalic acid, which has great application potential and economic value.^[^ ^4^ ^]^ Recently, due to the advantages of normal temperature and atmospheric pressure operating conditions, the attention to electrocatalytic HMF conversion has gradually increased.^[^ ^5^ ^]^ Benefiting from the abundant metal sites in the laminates, layered double hydroxides (LDHs) exhibit a good development potential in the field of HMF oxidation.^[^ ^6^ ^]^ However, limited by the weak intrinsic properties, the catalytic performance of the material is low, which still has wide room for improvement.^[^ ^7^ ^]^ Therefore, adopting appropriate modifications to further improve the catalytic oxidation activity of HMF has become a hot topic in this field.^[^ ^8^ ^]^

Defect engineering can adjust the degree of electron localization, change the band structure of the catalyst, and affect the adsorption behavior of reactants and intermediates, which is considered an ideal way to improve the catalytic activity of materials.^[^ ^9^ ^]^ To date, researchers have developed some catalysts rich in oxygen vacancies, which exhibit increased adsorption capacity and enhanced activity.^[^ ^10^ ^]^ In fact, according to the Sabatier principle, the optimal catalyst should maintain a moderate adsorption strength with the reactive species to balance the relationship between adsorption coverage and product desorption.^[^ ^11^ ^]^ Although the introduction of oxygen vacancy increases the adsorption capacity of the reactants and improves the catalytic performance, over‐strong adsorption also inhibits the desorption of the products, resulting in limited optimal expression of the activity. Therefore, how further modulating the oxygen vacancy to balance the adsorption behavior of the reactants is the key to achieving optimal performance, which is rarely involved in electrocatalytic biomass conversion.

Defect site filling, which is able to improve the oxygen evolution reaction (OER) performance, maybe a suitable method.^[^ ^12^ ^]^ On the one hand, the heteroatom filling at the defect site may compensate for part of the missing coordination number, weakening the over‐strong adsorption caused by excessive oxygen vacancy.^[^ ^13^ ^]^ On the other hand, suitable heteroatomic modifications may further optimize the charge transfer behavior of the material, and accelerate the catalytic reaction process.^[^ ^14^ ^]^ However, defect filling can also further break the symmetry by introducing heteroatoms, thus affecting the interaction with the reactants. Therefore, there are contradictions in the regulation of adsorption behavior by defect filling, which deserves further investigation. Notably, the regulation mechanism of defect site heteroatom filling in the electrocatalytic conversion of biomass is rarely mentioned, which is also worth further exploration.

Based on the above viewpoints, we designed and constructed an oxygen defect‐enriched CoFe─LDH, which contains Co elements with both direct and indirect oxidation properties and introduced heteroatom S for defect filling (S─Vo─LDH). The physical characterization and theoretical calculation results show that the introduction of S partially fills the oxygen vacancy in the second shell and modulates the d band structure by forming metal─S bond. The filling of S weakens the strong adsorption caused by excess oxygen vacancies and forms new adsorption sites to avoid the blockage of reaction sites caused by competitive adsorption at the same site. Benefiting from the optimized adsorption behavior of HMF and OH species caused by defect site S filling, the acceleration of interfacial charge transfer behavior, and the rapid formation of high‐valence catalytic sites in the reaction, this material exhibits excellent HMF oxidation activity compared with pure LDH, including a greatly advanced onset potential and a four‐fold higher current density at 1.4 V. This work deeply analyzes the enhancement mechanism of defect filling, fills the gap of defect filling strategy in the field of biomass electrocatalytic conversion and provides constructive guidance for the design and development of efficient catalysts in the future.

## 2 Results and Discussions

**Figure 1:**
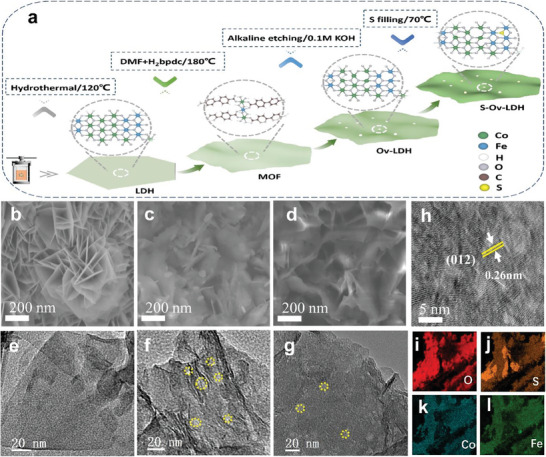
a) Schematic illustration of the synthesis of the S─Ov─LDH; SEM images of the b) LDH, c) Ov─LDH and d) S─Ov─LDH; TEM images of the e) LDH, f) Ov─LDH and g) S─Ov─LDH; h) HRTEM image of the S─Ov─LDH; i‐l) EDX Mapping images of the S─Ov─LDH.

S─Vo─LDH was prepared by a multi‐step hydrothermal‐etching‐ion modification method, which was given in **Figures** **1**a, S1 (Supporting Information). First, pure CoFe─LDH was synthesized by hydrothermal method, which was subsequently used as a precursor to growing CoFe metal–Organic framework (CoFe MOF, Figure S2, Supporting Information) material by in situ transformation. The obtained MOF was then immersed in KOH solution to convert to defective LDH (Vo─LDH). Finally, the above material was modified with sulfur by water bath thermal decomposition to prepare the sample filled with S ions at the defect sites, which was defined as S─Vo─LDH. As shown in Figure 1b–d and Figure S3 (Supporting Information), scanning electron microscopy (SEM) gives the difference in the morphological structure of the three different samples. The LDH nanosheets (Figure 1b; Figure S3a, Supporting Information) are grown vertically on the Ni foam substrate, displaying a smooth surface with a lateral size of 300–800 nm. After alkali etching and the introduction of S species, the surface of the S─Vo─LDH sample (Figure 1d; Figure S3c, Supporting Information) becomes rough and the structure is distorted, which is similar to the morphology of the defective Vo─LDH sample (Figure 1c; Figure S3b, Supporting Information) and completely different from pure LDH. To further elucidate the effects of etching and introduction of S species on the microstructure, transmission electron microscopy (TEM) was performed.^[^ ^15^ ^]^ Obviously, compared with the intact surface of pure LDH (Figure 1e; Figure S3d, Supporting Information), many holes appear on the surface of Vo─LDH (Figure 1f; Figure S3e, Supporting Information) and S─Vo─LDH (Figure 1g; Figure S3f, Supporting Information), indicating the successful construction of defective structures, which corresponds to the SEM results. Moreover, the lattice fringe corresponding to the (012) crystal face of LDH material can be observed by high‐resolution TEM (HRTEM, Figure 1h), which means that the LDH structure is still maintained after the defect filling. The result of selected area electron diffraction (SAED, inside in Figure S3d–f, Supporting Information) also shows the related crystal planes attributed to LDH structure, which further confirms this conclusion. In addition, the energy dispersive X‐ray Mapping spectrum (EDX‐Mapping) is also given in Figure 1i–l, representing the successful introduction of the S element and the uniform dispersion of multiple elements in the S─Vo─LDH sample.

**Figure 2:**
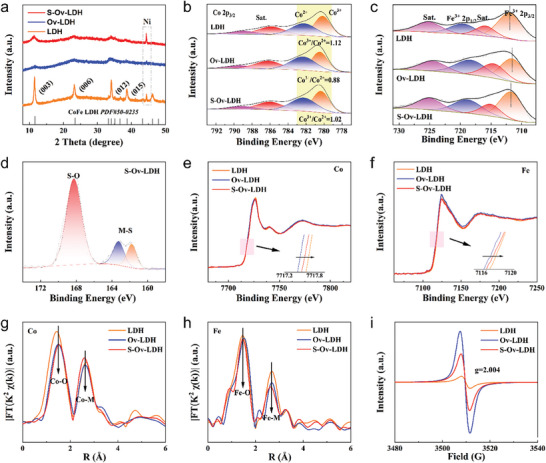
a) The XRD patterns of the LDH, Ov─LDH, and S─Ov─LDH samples; b, c) Co 2p, Fe 2p XPS spectra of the LDH, Ov─LDH, and S─Ov─LDH samples; d) S 1s spectrum of the S─Ov─LDH; e, f) Co *K*‐edge, Fe *K*‐edge XANES spectra of the LDH, Ov─LDH and S─Ov─LDH samples; g, h) EXAFS spectra in *R* space of the Co and Fe elements; i) EPR spectrum of the LDH, Ov─LDH and S─Ov─LDH samples.

Subsequently, the crystal structure of the sample was characterized by X‐ray powder diffraction (XRD). As shown in **Figure** **2**a, the diffraction peaks located at ≈11.5°, 23.1°, 34.1°, and 38.6° correspond to the (003), (006), (012), and (015) crystal planes of LDH structure.^[^ ^16^ ^]^ After defect regulation, the peak strength of the Vo─LDH material is weakened and no new peaks appear, which means that the LDH crystalline phase structure of the material is maintained and the symmetry is broken after modification. With the further filling of S species, the intensity of the characteristic peak is increased but still weaker than that of pure LDH, indicating that the unsaturated coordination structure formed by the construction defect is partially compensated. Notably, the peak at 44.5° is the characteristic peak of Ni, which is caused by scraping the powder off the Ni foam. X‐ray photoelectron spectroscopy (XPS, Figure S4, Supporting Information) was then employed to investigate the effect of defect site filling on the surface chemical environment of different materials. The high‐resolution Co 2p_3/2_ spectra can be deconvoluted into four subpeaks, including two core peaks and two corresponding satellite peaks.^[^ ^17^ ^]^ Among them, the characteristic peaks of Co^3+^ and Co^2+^ at 780.17 and 782.25 eV are observed for LDH, accompanied by the Co^3+^/Co^2+^ ratio of 1.12. In contrast, the Co^3+^/Co^2+^ ratios of Ov─LDH and S─Ov─LDH are 0.88 and 1.02, respectively (Figure 2b), which suggests that the valence state of Co element decreases and then slightly increases, corresponding to the formation and partial filling of oxygen vacancies. Similarly, the shift of the main peak is observed in the Fe 2p_3/2_ XPS spectra, the binding energies of Fe^3+^ for the three samples are 712.03, 711.55, and 711.73 eV respectively (Figure 2c), which implies that the trend of the Fe valence state is in agreement with that of the Co. On the other hand, the presence of a large number of oxygen vacancies leads to a negative shift of 0.36 eV in the M─O XPS peak position of Ov─LDH, while the partial filling of oxygen vacancies by S element leads to a slight positive shift of 0.13 eV (Figure S5, Supporting Information), which is also consistent with the above conclusions. For S species, three peaks clearly visible in the S 2p spectra are attributed to the S─O bond, and the 2p_1/2_ and 2p_3/2_ orbital splitting peaks of the metal─S bond (Figure 2d), indicating that S partially fills the oxygen vacancy by bonding with the metal. The relevant element contents are also given in Table S1, Supporting Information.

X‐ray absorption fine spectroscopy (XAFS, Figures S6–S12, Supporting Information) was further carried out to research in depth the electronic interactions and coordination environment. As shown in Figure 2e, the X‐ray absorption near edge structure (XANES) of Co element for Ov─LDH, S─Ov─LDH, and LDH along the arrow pointing to a gradual increase in binding energy, indicating that the construction of oxygen defects can reduce the valence state of Co, while the filling of S can compensate part of the oxygen vacancies, thus increasing the valence state again. The same change trend is also observed for the valence states of Fe species in the near‐edge structure (Figure 2f), which is consistent with the above viewpoint. The extended X‐ray absorption fine structure (EXAFS) fitting results show that the oxygen defect can destroy the symmetry of the material and cause the local coordination unsaturated, resulting in the coordination number of the Ov─LDH sample being significantly lower than that of the LDH sample (Tables S2 and S3, Supporting Information). In addition, the Co─S bond exists in S─Ov─LDH sample and is located in the second shell, indicating that S atoms affect the central atoms at a relatively far distance rather than at the original O atom position. Moreover, the Co─Co bond length in S─Ov─LDH sample is significantly shorter than that in Ov─LDH and LDH, further suggesting that the formation of Co─S bond in the second shell has a significant impact on the electronic structure (Figure 2g). For Fe species, the average coordination number of Fe─O and Fe─Fe changes with the construction of defects and the filling of S, and the Fe─S bond structure also appears in the second shell of S─Ov─LDH sample, which further supports the above conclusions (Figure 2h). Subsequently, the oxygen vacancy content of different catalysts was visualized using electron paramagnetic resonance (EPR, Figure 2i, Figure S13, Supporting Information) spectroscopy. After the introduction of the S element, the oxygen vacancy signal of the S─Ov─LDH catalyst is significantly weakened but still stronger than that of pure LDH, which also strongly proves that the oxygen vacancy is partially filled by S atoms, further supporting the above views. The specific defect concentrations are given in Figure S13 (Supporting Information). In addition, Raman spectra were employed to characterize the molecular structure information of different samples. As shown in Figure S14 (Supporting Information), the three samples show the same LDH characteristic peaks at ≈450 and ≈520 cm^−1^, corresponding to the M─O(H) and M─O vibrations, respectively. Notably, the variation trend of the peak strength of the three samples is consistent with the results of XRD and EPR, which further indicates the formation and partial filling of defects and disordered structures. Furthermore, the calculated d‐band center positions from XPS valence band spectra indicate that the d‐band centers of LDH, S─Ov─LDH, and Ov─LDH gradually approach the Fermi level (Figure S15, Supporting Information), implying that defect construction can promote the interaction between reactants and catalysts, while defect filling can weaken this enhanced interaction to some extent, further clarifying the regulation effect of defect filling.

**Figure 3:**
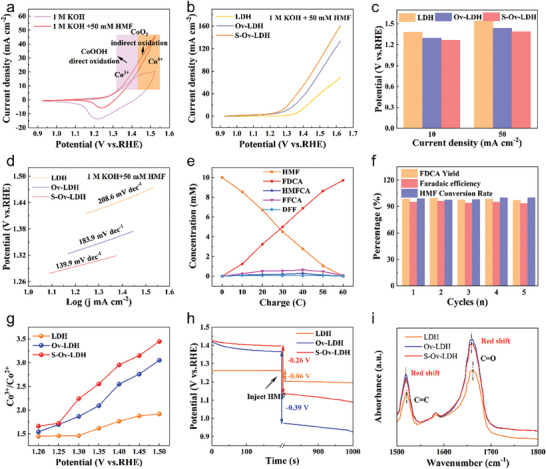
a) CV curves of the LDH, Ov─LDH and S─Ov─LDH samples; b) LSV polarization curves of the LDH, Ov─LDH and S─Ov─LDH samples; c) the corresponding relationship between current density and potential of the above three samples; d) the corresponding Tafel plots of the LDH, Ov─LDH and S─Ov─LDH samples; e) the concentration of substrates, intermediates, and products during the HMFOR for S─Ov─LDH; f) Faradaic efficiency and FDCA yield of S─Ov─LDH under five successive electrolysis cycles; g) Ratio of Co^3+^/Co^2+^ at different potentials for the above three samples; h) OCP changes of the three samples before and after HMF injection in KOH environment; i) ATR‐FTIR spectrum of different samples after adsorption of HMF molecule.

The HMF electrocatalytic oxidation performance of different samples was evaluated under alkaline conditions (1 m KOH with 50 mm HMF, Figure S16, Supporting Information). Cyclic voltammetry (CV) curves were employed to identify the oxidation type of the material in the reaction. In the KOH environment, the oxidation peaks located at 1.15 and 1.43 V correspond to the oxidation of Co^2+^→Co^3+^ and Co^3+^→Co^4+^, respectively.^[^ ^18^ ^]^ As shown in **Figure** **3**a, the oxidation current density of the material in the HMF+KOH solution is higher than that in the KOH environment within the potential range of 1.32‐1.43 V, indicating that the direct oxidation of HMF is controlled by Co^3+^ during this potential range. When the potential exceeds 1.43 V, the HMF oxidation current density of the material is still higher, which means that the indirect oxidation reaction involving Co^4+^ occurs after this potential. Figure 3b gives the linear sweep voltammetry (LSV) curves of the three samples. In contrast, S─Ov─LDH exhibits lower oxidation potential (1.26 V at 10 mA cm^−2^) and higher reaction current density (50 mA cm^−2^ at 1.39 V), which are even better than most related electrocatalysts (Figure S17 and Table S4, Supporting Information), indicating that the introduction of oxygen defects and further filling of S species can effectively improve the catalytic oxidation performance of the material. In order to more intuitively identify the differences in the performance of different samples, the relevant current density and reaction potential bar graphs are also shown in Figure 3c. Clearly, with the introduction of defects and the filling of S, the potential required to reach 10 mA cm^−2^ gradually decreases, and the current density reached at 1.35 V gradually increases, reflecting the promoting effect of defect construction and defect filling. In addition, the performance of pure Ni foam is also given in Figure S18, Supporting Information. The catalytic oxidation performance of Ni foam is significantly lower than that of LDH, Ov─LDH, and S─Ov─LDH materials, indicating that its primary role is to act as a carrier for the catalyst. After Brunauer‐Emmett‐Teller surface area test (BET) normalization (Figure S19, Supporting Information), S─Ov─LDH still exhibits the highest current density, implying its optimal intrinsic performance. In addition, as the Tafel slope of the sample (Figure 3d) decreases from 208 to 139 mV dec^−1^, representing the acceleration of the reaction kinetics, which further indicates the superiority of the defect‐filled catalyst. We have also conducted extended experiments on different substrates such as ethanol, cyclohexanol, cyclohexanone, furfuryl alcohol, furfural, benzylamine, etc. As shown in Figure S20 (Supporting Information), the S─Ov─LDH catalyst exhibits higher catalytic activity in a variety of nucleophile substrate oxidation reactions, demonstrating the universality of the material.

Product selectivity, yield, and Faraday efficiency are other important indicators for testing catalytic performance. High‐performance liquid chromatography (HPLC) test was performed for this purpose, and a more detailed description was shown in the product analysis section of supporting information (Figure S21, Supporting Information). As shown in Figure 3e, with the increase of coulomb amount, the substrate HMF is gradually consumed, and the product FDCA concentration is gradually increased. In this process, the main intermediate is 5‐hydroxymethyl‐2‐furancarboxylic acid (HMFCA) rather than 2,5‐dimethylfuran (DFF), which indicates that the oxidation process follows the HMF→HMFCA→FFCA→FDCA pathway under alkaline conditions (Figure S22, Supporting Information).^[^ ^19^ ^]^ Compared with pure LDH, S─Ov─LDH shows higher yield (98.4%), Faraday efficiency (94.9%), and substrate conversion (99.1%) (Figures S23 and S24, Supporting Information), which further supports the above viewpoints. After five electrolytic cycles, the FDCA yield of the S─Ov─LDH catalyst can still reach ≈97%, and the Faraday efficiency is likewise maintained at a high level of ≈94%, with no significant fluctuations in the metrics after each electrolysis, demonstrating excellent stability (Figure 3f). In addition, the microstructure and surface chemical environment of the material are basically unchanged after the reaction, which also confirms the above conclusions (Figures S25–S27, Supporting Information).

In order to explore the specific reasons for the enhanced catalytic performance of S─Ov─LDH, we obtained the Co^3+^/Co^2+^ ratio by quasi─Operando XPS test (Figure S28, Supporting Information), aiming to clarify the differences between different materials from the perspective of active sites. As shown in Figure 3g, the Co^3+^/Co^2+^ ratio of the three samples increases with the gradual increase of the applied potential. In contrast, the content of Co^3+^ in S─Ov─LDH is higher at the same reaction potential, which suggests that more catalytic sites in the material are involved in the HMF oxidation reaction process, resulting in a significant improvement of catalytic performance. We also investigated the valence change of the active site from the perspective of reaction kinetics. According to the pseudo‐capacitance principle, the charge and discharge time of transition metal‐based electrode materials in the high potential region is controlled by the redox of the active site in the reaction region. Obviously, S─Ov─LDH exhibits a longer charge and discharge time (Figure S29, Supporting Information), reflecting that defect site filling can effectively promote the valence state transformation of the material in the reaction, which further supports the above viewpoints. In addition, adsorption is the first step in a catalytic reaction and is a significant factor that affects catalytic activity. Open‐circuit potential (OCP) tests were performed to distinguish the variation of organic adsorbed species in the inner Helmholtz layer of different materials.^[^ ^20^ ^]^ As shown in Figure 3h, the OCP change of Ov─LDH is significantly greater than that of LDH after HMF molecule injection, indicating that the construction of defects is conducive to the adsorption of organic species. After the further introduction of S, the change of OCP is reduced, but still greater than LDH, suggesting that defect filling can effectively balance the adsorption effect of organic species. Attenuated Total Refraction‐Fourier Transform Infrared spectroscopy (ATR‐FTIR) was also employed to characterize the adsorption behavior of organic substrate on the surface of materials. Compared with pure LDH, Ov─LDH and S─Ov─LDH exhibit significantly blue‐shifted absorption peak positions of C═O and C═C functional groups and higher absorption peak intensity (Figure 3i), which means that HMF molecules have higher coverage on the surface of these two samples. However, the peak intensity of S─Ov─LDH is slightly lower than that of Ov─LDH, indicating that the filling of S weakens the adsorption enhancement effect of oxygen defects on organic molecules to a certain extent, which is also consistent with the above OCP results.

**Figure 4:**
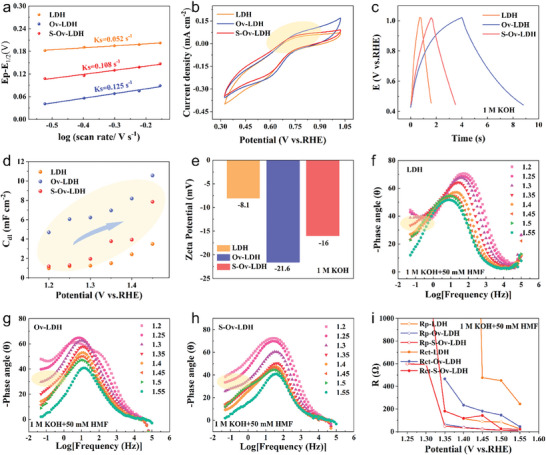
a) Ks values of the LDH, Ov─LDH, and S─Ov─LDH samples; b) CV curves of the LDH, Ov─LDH, and S─Ov─LDH samples; c) the charge and discharge curves of the three samples in the low potential range; d) the *C*_dl_ values of the above three samples; e) Zeta potential of the LDH, Ov─LDH, and S─Ov─LDH samples; f–h) the Bode plots of the LDH, Ov─LDH, and S─Ov─LDH samples; i) *R*_p_ and *R*_ct_ values of the above three samples.

In addition to the adsorption of organic substrates, the cobalt‐based active electrocatalyst also should have an appropriate affinity for the OH species. We also described the differences in OH adsorption and conversion of different materials from various aspects to analyze the catalytic enhancement effect of defect filling. According to the Laviron equation, the redox potential difference has a linear relationship with the logarithm of the sweep speed within a certain range, indicating that the redox rate‐limiting step of the transition metal site is controlled by the mass diffusion of OH^−^ ions from the electrolyte to the electrode surface and the coupling with the metal site.^[^ ^21^ ^]^ As shown in **Figure** **4**a and Figures S30–S32 (Supporting Information), Ov─LDH (Ks = 0.125 s^−1^) exhibits a higher redox constant than LDH (Ks = 0.052 s^−1^) due to the introduction of oxygen defects, implying that the former possesses a higher binding capacity of OH species. However, too strong OH adsorption may lead to excessive occupation of the active site, thus forming a competitive adsorption relationship with the organic catalytic substrate, which is not conducive to the maximization of catalyst efficiency. It is worth noting that after the introduction of the S element, the Ks value of S─Ov─LDH catalyst drops to 0.108 s^−1^, indicating that the filling of defect sites by S effectively compensates for this weakness. Besides, the CV curve at the low potential interval was also performed to further determine the adsorption and conversion ability of the material to OH species from the dynamics.^[^ ^22^ ^]^ The adsorption peak intensity of S─Ov─LDH is between Ov─LDH and LDH, indicating a moderate OH^−^ adsorption and conversion capacity (Figure 4b), which is consistent with the above results. Whether HMF is added or not, the results of charging and discharging in this potential range are also consistent with the above trend, which further supports this inference (Figure 4c; Figure S33, Supporting Information). On the other hand, we also analyzed the difference in the adsorption behavior of OH^−^ during the reaction process from the perspective of capacitance. The capacitance of Ov─LDH in the positive potential reaction region is significantly higher than that of pure LDH (Figure 4d), indicating that the construction of defect sites greatly improves the enrichment effect of the material on OH^−^. However, the introduction of S element weakens the capacitance value, which confirms the regulating effect of defect filling on the adsorption behavior of OH^−^ species. Moreover, the Zeta potential in the alkaline environment, which represents the enrichment of OH^−^ ions in the inner Helmholtz layer, was also performed.^[^ ^23^ ^]^ As shown in Figure 4e, the value of S─Ov─LDH is about twice that of LDH and lower than that of Ov─LDH, further reflecting its moderate OH adsorption capacity. Furthermore, we also analyze the promotion effect of defect filling from the perspective of adsorption oxygen ratio. The XPS results show that the adsorption oxygen ratio of S─Ov─LDH is between the other two materials, which also indicates the moderate adsorption capacity of this material for OH species, confirming the above conclusions (Figure S34, Supporting Information).

The above section explores in detail the regulation effect of defect filling on the adsorption equilibrium of organic substrate HMF and OH species. In order to further investigate the effect of defect filling on interfacial charge transfer dynamics, in situ electrochemical impedance spectroscopy (in situ EIS) was employed.^[^ ^24^ ^]^ Figure 4f–h gives the Bode plots of the HMF oxidation reaction (HMFOR) process for LDH, Ov─LDH, and S─Ov─LDH at 1 m KOH and 50 mm HMF solution. The surface oxidation of the catalyst and the HMFOR process occurs in high‐frequency regions and low‐frequency regions, respectively. Compared with LDH and Ov─LDH, the inflection point potential of S─Ov─LDH in the low‐frequency region is lower, indicating that HMF oxidation can occur at a lower potential. Moreover, the phase angle of S─Ov─LDH in the low‐frequency region of the reaction interval is much lower than that of Ov─LDH and LDH, further indicating its faster HMF oxidation reaction kinetics (Figure 4f–g). Subsequently, by selecting the optimal fitting model, we further analyze the electrode internal oxidation and electrode interface reaction of different materials (Figure S35, Supporting Information). *R*_p_ and *R*_ct_ represent the internal impedance of the electrode and the electrode‐electrolyte interface charge transfer resistance, respectively.^[^ ^25^ ^]^ The specific values are shown in Figure 4i. For S─Ov─LDH, we observe that *R*_p_ and *R*_ct_ values are lower than LDH and Ov─LDH at almost all measured potentials, indicating that partial filling defect sites with S elements are favorable for charge transfer dynamics in both internal oxidation of electrodes and electrooxidation of HMF (Figure S36, Supporting Information). On the other hand, the OER potential of S─Ov─LDH is ≈1.45 V, which can be found in Bode plots and Nyquist plots, and is lower than that of pure LDH samples (Figures S37–S39, Supporting Information). In order to avoid the occurrence of competitive OER, the optimal HMF oxidation reaction potential should be lower than 1.45 V.

**Figure 5:**
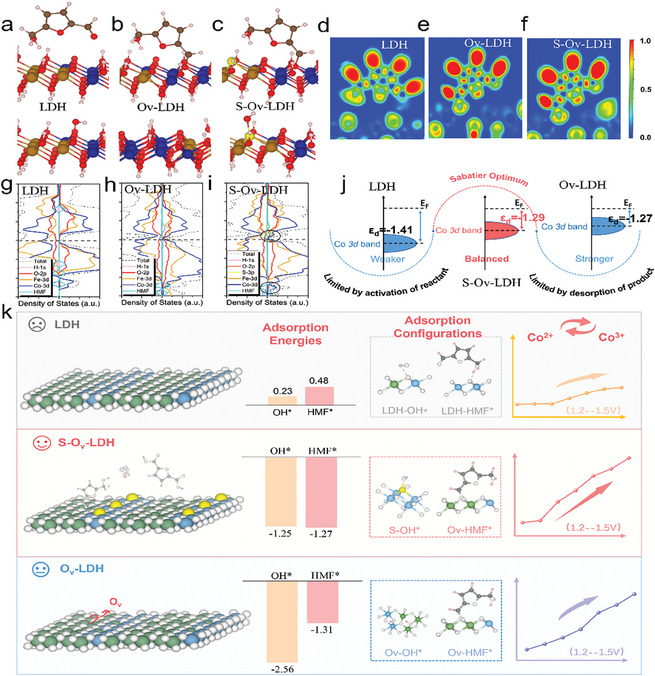
Adsorption configurations of HMF and OH on a) LDH, b) Ov─LDH and c) S─Ov─LDH; ELF plots of the d) LDH, e) Ov─LDH and f) S─Ov─LDH; the PDOS spectra of the g) LDH, h) Ov─LDH and i) S─Ov─LDH; j) the d band center spectrum of the above three samples; k) the diagram of catalytic enhancement mechanism of S─Ov─LDH.

To unravel the high‐activity origin of S─Ov─LDH toward HMFOR, density functional theory (DFT) calculations were then conducted to obtain atomic‐level information.^[^ ^26^ ^]^ First, the structure models of LDH, Ov─LDH, and S─Ov─LDH were constructed based on the experimental results, and the optimized adsorption configurations of HMF on these models are shown in **Figure** **5**a–c. It is observed that HMF can only be physically adsorbed on LDH, while the formyl group of HMF is chemically adsorbed on the oxygen vacancy of S─Ov─LDH and Ov─LDH. As shown in Figure S40 (Supporting Information), the optimal adsorption energies (Eads) for HMF on LDH, Ov─LDH, and S─Ov─LDH are 0.48,1.31, and1.27 eV, which suggests that the interaction between HMF and the catalyst gets stronger with the introduction of oxygen vacancy but weakens with the filling of oxygen vacancy by S, and finally reaches the optimal state. This conforms to the Sabatier principle, which states that moderate adsorption energy helps to reduce the reaction barrier and accelerate the HMFOR process. A similar trend is observed for OH adsorption (Figure S40, Supporting Information). These results suggest that proper S filling of oxygen vacancies can weaken the over‐adsorption of reactants induced by excessive oxygen vacancies on the basis of enhancing the intrinsic adsorption capacity, thus triggering the appropriate adsorption of reactants and intermediates and improving the catalytic activity during the HMF oxidation process. Notably, unlike the adsorption of HMF at the oxygen vacancy site, the OH adsorption site of S─Ov─LDH is S species, which is undoubtedly conducive to avoiding excessive competitive adsorption of two reactants at the same site, and promotes the oxidation process from the perspective of reaction site distribution.

In addition, based on the charge density difference calculations, the electron transfer from the catalysts to HMF could be revealed (Figure S41, Supporting Information). The electron transfer number on S─Ov─LDH increases and then decreases with the introduction of oxygen vacancy and partial filling of S species (0.01 e vs 0.11 e vs 0.08 e), indicating its moderately enhanced reactant activation. Electron‐localization function (ELF) plot is another powerful tool for analyzing the bonding type and electron distributions.^[^ ^27^ ^]^ As can be seen from Figure 5d–f, the ELF value at the connection point between S─Ov─LDH and HMF is higher than LDH but lower than Ov─LDH, indicating that the binding strength is in the middle value, which further verifies the adsorption regulation effect of defect filling. Besides, the projected density of states (PDOS) in Figure 5g–i shows that the obvious orbital hybridization around the Fermi level for Ov─LDH, S─Ov─LDH, and HMF also indicates a strong bonding cooperation between them induced by defect engineering.^[^ ^28^ ^]^ The total density of states (TDOS) plots at the Fermi level demonstrate the improved electron state density and optimal charge transfer effect of the S─Ov─LDH in comparison with both LDH and Ov─LDH (Figure S42, Supporting Information).^[^ ^29^ ^]^ Moreover, the calculated d band center also conforms to the above trend and is consistent with the results of the valence band spectrum mentioned above, which further verifies this view. Notably, due to the differences in conditions between theory and experiment, the specific values calculated by the two methods are not the same. In this regard, benefiting from the defect‐filling strategies (Figure 5k), S─Ov─LDH presents increased catalytic sites, optimal conductivity, separated adsorption sites, and moderate adsorption energy (moderate chemical interaction with HMF and OH^−^), which is conducive to the efficient HMFOR process.

## 3 Conclusion

In conclusion, in order to further regulate the HMF catalytic performance of LDH, the S─Ov─LDH catalyst was designed and constructed by defect filling strategy, which exhibited lower reaction potential (1.26 V at 10 mA cm^−2^) and four times higher catalytic current density at 1.4 V than pure LDH. A variety of physical characterizations, electrochemical tests, and theoretical calculations confirm that the S element filling at the defect site can affect the electronic structure in the second shell through the formation of metal─S bonds, thus promoting the formation of high‐valence Co^3+^ active species during the reaction, weakening the over‐adsorption of reactants induced by excessive oxygen vacancies, regulating the adsorption equilibrium of the reactive molecules, and forming new adsorption sites to promote the reactant transformation. This work analyzes the regulation effect of defect filling on the structure and catalytic performance of materials in detail, fills the gap of defect filling strategy in the field of biomass upgrading, and provides constructive guidance for the design and development of efficient catalysts in the future.

## Conflict of Interest

The authors declare no conflict of interest.

## Supporting information



Supporting Information

## Data Availability

The data that support the findings of this study are available from the corresponding author upon reasonable request.;
