# Supplementary material for: Oxygen Defect Site Filling Strategy Induced Moderate Enrichment of Reactants for Efficient Electrocatalytic Biomass Upgrading
Source: Adv Sci (Weinh). 2024 Nov 4;11(48):2410725. doi: 10.1002/advs.202410725 (PMC11672306; doi:10.1002/advs.202410725)
Supplement: Supplementary file 1 — Supporting Information [file ADVS-11-2410725-s001.pdf]

ADVANCED SCIENCE
Open Access

# Supporting Information

for *Adv. Sci.*, DOI 10.1002/advs.202410725

Oxygen Defect Site Filling Strategy Induced Moderate Enrichment of Reactants for Efficient Electrocatalytic Biomass Upgrading

*Baixue Cheng, Haoyu Zhan, Yankun Lu, Danning Xing*, Xingshuai Lv*, Thomas Frauenheim, Peng Zhou*, Shuangyin Wang and Yuqin Zou**

# Supporting Information

**Oxygen Defect Site Filling Strategy Induced Moderate Enrichment of Reactants for Efficient Electrocatalytic Biomass Upgrading**

*Baixue Cheng,[a] Haoyu Zhan,[a] Yankun Lu,[a] Danning Xing,[c*] Xingshuai Lv,[d]* Thomas Frauenheim,[e,f,g] Peng Zhou,[a]* Shuangyin Wang,[b] Yuqin Zou,[b]**

Prof. P. Zhou, Dr. B. Cheng, Dr. H. Zhan, Dr. Y. Lu
a: State Key Laboratory of Bio-fibers and Eco-textiles, College of Materials Science and Engineering, Collaborative Innovation Center of Shandong Marine Biobased Fibers and Ecological Textiles, Institute of Marine Biobased Materials, Qingdao University, Qingdao 266071, P. R. China
E-mail: pengzhou@qdu.edu.cn

Prof. S. Wang, Prof. Y. Zou
b: State Key Laboratory of Chemo/Bio-Sensing and Chemometrics, College of Chemistry and Chemical Engineering, Advanced Catalytic Engineering Research Center of the Ministry of Education, Hunan University, Changsha, 410082, China
E-mail: yuqin_zou@hnu.edu.cn

Prof. D. Xing
c: Shandong Institute of Advanced Technology, Jinan 250100, P. R. China
E-mail: danning.xing@iat.cn

Prof. X. Lv
d: College of Chemistry and Chemical Engineering, Ocean University of China, Qingdao 266100, P. R. China
E-mail: lvxs@ouc.edu.cn

Prof. T. Frauenheim
e: School of Science, Constructor University, Bremen 28759, Germany
f: Beijing Computational Science Research Center, Beijing 100193, China
g: Institute for Advanced Study, Chengdu University, Chengdu 610106, China

**Experimental Section**

**Materials**

Cobalt(II) nitrate hexahydrate ($Co(NO_3)_2·6H_2O$, ≥98.5%), iron(III) nitrate nonahydrate ($Fe(NO_3)_3·9H_2O$, ≥98.5%), urea ($CH_4N_2O$, ≥99.5%), ammonium fluoride (NH4F, ≥96%), potassium hydroxide (KOH, ≥85%), sodium thiosulfate pentahydrate ($Na_2S_2O_3·5H_2O$, ≥99%), N,N-dimethylformamide (DMF, $C_3H_7NO$, ≥99.5%), biphenyl-4,4′-dicarboxylic acid ($H_2$bpdc, $C_{14}H_{10}O_4$, ≥97%), acetone ($C_3H_6O$, 99.5%), hydrochloric acid (HCl, AR), 5-hydroxymethylfurfural (HMF, $C_6H_6O_3$, 96%), 2,5-furandicarboxylic acid (FDCA, $C_6H_4O_5$, 97%), furan-2,5-dicarbaldehyde (DFF, $C_6H_4O_3$, 98%), 5-hydroxymethyl-2-furancarboxylic acid (HMFCA, $C_6H_6O_4$, 98%), 5-formyl-2-furancarboxylic acid (FFCA, $C_6H_4O_4$, 98%), ammonium formate ($CH_5NO_2$). All chemicals used commercially purchased without further purification.

**Synthesis of layered double hydroxide (LDH) catalysts**

Nickel foam (NF) was submerged in 2 M HCl and sonicated for 15 min, and then treated with acetone and ethanol in the above steps in sequence to remove surface oxides and impurities to obtain a clean nickel foam. The obtained NF was vacuum-dried at 60 ℃ overnight.

30 ml deionized water was added into a Teflon liner (50 mL) containing $Co(NO_3)_2·6H_2O$, $Fe(NO_3)_3·9H_2O$ (molar ratio is 3:1 and the total molar amount is 5 mmol), urea (4.33 mmol), $NH_4F$ (2.16 mmol) to mix evenly, and then the clean 2 × 3 $cm^2$ piece of NF was tilted into the solution. It was heated at 120 ℃ for 6 h in a stainless-steel autoclave. After the reaction, the solution was cooled to room temperature, the surface of the NF was carefully washed with deionized water and ethanol three times respectively, and then placed in 60 ℃ vacuum drying overnight to obtain LDH.

**Synthesis of defective LDH (Ov-LDH) catalysts**

With the aid of sonication, 0.50 mmol of $H_2$bpdc was dispersed in 30 mL of DMF to obtain solution A. Solution A was then transferred to a 50 mL Teflon liner. Subsequently, LDH was tilted into the liner and heated in a stainless steel autoclave at 180 °C for 12 h to obtain CoFe metal-organic framework (CoFe MOF), which was washed three times with DMF and ethanol and then dried in an oven at 60 °C overnight. The CoFe MOF was placed in a 0.1 M KOH aqueous solution and left at room temperature for 6 h until it was completely converted to Ov-

LDH. finally, it was again washed three times by deionized water and ethanol and dried in an oven at 60 °C overnight.

**Synthesis of S filled Ov-LDH (S-Ov-LDH) catalysts**

The cleaned Ov-LDH was immersed in 0.1 M $Na_2S_2O_3$ solution and reacted for 30 min at 70 °C. Before use, the product was completely rinsed with deionized water and ethanol, dried overnight at 60 °C, and labeled as S-Ov-LDH.

**Characterization**

The surface morphology and compositional structure of the samples were observed by a field emission scanning electron microscope (SEM, Zeiss, US) model sigma 500. The internal fine structure of the sample was characterized by a transmission electron microscope (TEM, JEOL, Japan) model 2100 plus. X-ray photoelectron spectroscopy was performed on an ESCALAB Xi+ electron spectrometer (XPS, Thermo Fisher Scientific, US) using 300 W Al Kα radiation. Energy calibration was carried out using the C 1s peak of adventitious C at 284.8 eV. The adsorption characterization was done by Fourier transform infrared spectroscopy, (FTIR, Nicolet iN10MX, Thermo Fisher Scientific, US) and DLS-Zeta potential (Nano ZSE, Malvern, UK). FTIR test method: The sample was immersed in HMF solution for 5 min, and then dried in an oven at 120 ℃ for 3.5 h to obtain the sample to be tested. Then the IR device was adjusted to ATR mode. The obtained material was placed on the sample stage and the test procedure was initiated to obtain the FTIR spectra. X-ray diffraction (Smart Lab 3KW, Rigaku, Japan) is used to study the crystal structure of materials. Raman spectrum was performed using Thermofisher DXR2 Raman Spectrometer. Specific surface area and isothermal adsorption curves of the samples were obtained by a fully automated specific surface and porosity analyzer (ASAP 2460, Micromeritics instrument Ltd. America). A contact angle/surface tension meter (Dataphysics OCA20, German) is used to characterize the hydrophilicity of the material. To determine the concentration of oxygen defects of the samples, electron paramagnetic resonance (EPR) spectra were taken on a Bruker EMX plus, (German) continuous-wave EPR spectrometer by applying an X band (9.8 GHz) and a sweeping magnetic field at room temperature. According to the material number, the sample was loaded into a quartz tube, and then the quartz tube was placed in the resonance chamber of the instrument to ensure that the sample was located at the center of the chamber. And then the scanning test was started. Laser micro-Raman spectroscopy (DXR2, Thermo Fisher Scientific, US), on the other hand, serves as a complementary characterization for defect detection. Inductively coupled plasma atomic

emission spectroscopy (ICP-OES) was performed on a PerkinElmer 8300 ICP optical emission spectrometer (PerkinElmer, Shanghai). Extended X-ray absorption fine structure (EXAFS) and X-ray absorption near edge structure (XANES) of the K-edge of the sample were measured on a laboratory device (easyXAFS300, easyXAFS LLC), which is based on Rowland circle geometries with spherically bent crystal analyzers (SBCA) and operated using an Ag X-ray tube source and a silicon drift detector (AXAS-M1, KETEK GmbH). All spectra were collected under ambient conditions. Data reduction, data analysis, and EXAFS fitting were performed and analyzed with the Athena and Artemis programs of the Demeter data analysis packages (reference 1： B. Ravel and M. Newville, ATHENA, ARTEMIS, HEPHAESTUS: data analysis for X-ray absorption spectroscopy using IFEFFIT, Journal of Synchrotron Radiation 12, 537–541 (2005)) that utilizes the FEFF6 program (reference 2： Zabinsky, S. I.; Rehr, J. J.; Ankudinov, A.; Albers, R. C.; Eller, M. J. Multiple-Scattering Calculations of X-Ray-Absorption Spectra. Phys. Rev. B 1995, 52 (4), 2995−3009.) to fit the EXAFS data. The energy calibration of the sample was conducted through standard and Co foil, which as a reference was simultaneously measured. A linear function was subtracted from the pre-edge region, then the edge jump was normalized using Athena software. The χ(k) data were isolated by subtracting a smooth, third-order polynomial approximating the absorption background of an isolated atom. The k3-weighted χ(k) data were Fourier transformed after applying a HanFeng window function (Δk = 1.0). For EXAFS modeling, the global amplitude EXAFS (CN, R, σ2 and ΔE0) were obtained by nonlinear fitting, with least-squares refinement, of the EXAFS equation to the Fourier-transformed data in R-space, using Artemis software, EXAFS of the Co foil are fitted and the obtained amplitude reduction factor S02 value (0.762) was set in the EXAFS analysis to determine the coordination numbers (CNs) in sample.

The k-space and r-space values of Co and Fe elements for LDH sample are 2.5-9 (Co k-space), 2.46-9.674 (Fe k-space) and 1.1-3 (Co r-space), 1-3.1 (Fe r-space), respectively. The k-space and r-space values of Co and Fe elements for Ov-LDH sample are 2-8.5 (Co k-space), 2.46-9.286 (Fe k-space) and 1.13-3 (Co r-space), 1.1-3 (Fe r-space) respectively. The k-space and r-space values of S-Ov-LDH sample are 2-8.5 (Co k-space), 2.46-9.951 (Fe k-space) and 1.13-4 (Co r-space), 1-3 (Fe r-space) respectively.

**Calculation Methods**

All spin-polarized density functional theory (DFT) calculations were implemented by using the Vienna *ab initio* simulation package (VASP)[1]. The projector-augmented wave (PAW) method[2] and the Perdew−Burke−Ernzerh (PBE) functional[3] within the generalized

gradient approximation (GGA) were used in this work. The kinetic cutoff energy for the plane wave basis was set to 450 eV. The convergence threshold was set to $10^{-5}$ eV and 0.02 eV/Å for energy and force, respectively. Partial occupancies of the Kohn−Sham orbitals were allowed using the Gaussian smearing method and a width of 0.1 eV. Based on experimental TEM and XRD characterizations, CoFe-LDH (003) is the major exposed surface, which was used in these calculations where all atoms were then relaxed until convergence was reached. The basal plane of the CoFe LDH was constructed by using a 3×3×1 supercell, and oxygen atom was substituted by sulfur element with the consideration of oxygen defect. A ~15 Å vacuum layer was added along the *z*-axis to avoid spurious interactions of neighboring layers. The Brillouin zone was sampled by a 4 × 3 × 1 Γ-centered Monkhorst−Pack *k*-mesh for geometry optimizations. Grimme's DFT-D3 scheme[4] of dispersion correction was adopted to describe van der Waals (vdW) interactions between catalysts and adsorbates. Self-interaction errors for strongly correlated transition metal elements were corrected by using Hubbard U framework with Dudarev rotationally invariant method[5]. The U values were selected according to previous literatures[6,7]. The charge density difference (CDD)[8], projected density of states (PDOS), Bader charge analysis[9], and electron-localization function (ELF)[10] were calculated to assess the interaction strength.

The adsorption energy was defined as:

$$E_{\mathrm{ads}} = E_{\mathrm{total}} - E_{\mathrm{substrate}} - E_{\mathrm{adsorbate}}$$

where $E_{\mathrm{total}}$, $E_{\mathrm{substrate}}$, and $E_{\mathrm{adsorbate}}$ represent the total energy of the optimized adsorbate/substrate system, the clean substrate and the adsorbate in the gas phase, respectively.

**Electrochemical measurements**

The electrochemical properties of the resulting materials were measured using a standard three-electrode system in an alkaline environment. A Hg/HgO electrode was used as the reference electrode, a graphite rod as the counter electrode, and nickel foam with the prepared material attached as the working electrode. The overpotential, stability, change of potential and impedance of the electrodes were measured using electrochemical test methods such as cyclic voltammetry (CV), linear scanning voltammetry (LSV), timed amperometric curves (i-t), open-circuit potential (OCP) and electrochemical impedance spectroscopy (EIS), where impedance testing was done by Princeton electrochemical workstation, U.S.A. LSV tests were carried out in 1 M KOH (pH=13.8) solution or 1 M KOH with 50 mM HMF solution and tested using CHI760E electrochemical workstation. LSV tests were performed in an undivided cell, while

constant potential electrolysis tests were conducted in an H-type divided cell separated by a Nafion 117 membrane. LSV tests were performed at a scan rate of 5 mV $s^{-1}$, none of which were iR compensated. CV tests were carried out in different cyclic scans at a scanning rate of 100 mV $s^{-1}$, until the stable CV curves were obtained. The solution was continuously stirred at a rate of 500 rpm during the test. OCP test method: In the three-electrode system, the sample was placed in 1M KOH solution and the start button for the OCP mode was clicked for testing. After the voltage stabilized, the test was suspended, and 50 mM HMF was added to the solution and stirred at a constant speed. Then clicked the pause button again to continue the test and observed the voltage drop trend. EIS tests were performed in the frequency range of $10^{-2}$-$10^{5}$ Hz. The conversion equations are given below.

$V_{RHE} = 0.098 + 0.059 \times pH$

ZSimpWin software can provide the conversion of constant phase element (CPE) to capacitance (C) from the EIS data. Equivalent capacitance is as follows:

A CPE is represented by Q and its admittance Y is given by following formula:

$$Y=Y_0(j\omega)^n \quad (1)$$

j is the current density, ω is the angular velocity. For the R(QR) circuit, the admittance contribution from the (QR) term can be written as following formula:

$$Y=1/R[1+(j\omega Y_0^{1/n}R^{1/n})^n]=1/R[1+(j\omega\tau)^n] \quad (2)$$

where τ is the relaxation time constant. For the circuit with the CPE converted to a capacitor (R(CR)), admittance contribution from the (CR) term is:

$$Y=1/R(1+j\omega CR)=1/R(1+j\omega\tau) \quad (3)$$

The values of the resistance in the (QR) and (CR) are equal. To maintain the time constant,

$$\tau=CR=Y_0^{1/n}R^{1/n} \quad (4)$$

The capacitance for the CPE is given by $C=(Y_0R^{1/n})/R$ (5)

The specific values of R and n can be obtained by fitting. According to the above formulas, the capacitance value can be obtained from the ZSimpWin software.

*In-situ* EIS: The applied potential of the sample in 1M KOH solution ranged from 1.2 V to 1.65 V with an interval of 0.05 V. The relevant data were obtained by testing under different potentials. The applied potential of the sample in 1M KOH + 50 mM HMF solution ranged from 1.2 V to 1.55 V with an interval of 0.05 V. The relevant data were obtained by testing under different potentials.

Quasi-operando XPS: The samples were electrolyzed in 1M KOH + 50 mM HMF solution for 20 min at different potentials, respectively. The applied potential ranged from 1.2 V to 1.5

V with an interval of 0.05 V. After the electrolysis was completed, the sample were cleaned and dried, and the obtained samples were pressed for XPS testing.

**Products analysis**

In the study of this reaction system a 1260 InfinityII High Performance Liquid Chromatography (HPLC) equipped with ultraviolet-visible detector manufactured by Agilent, Germany was used for component analysis. The column used was Shim-pack GWS (5 µm, 4.6 × 150 mm). The specific analyzing conditions were as follows:

Column chamber temperature: 30°C.

Mobile phase speed: 0.6 ml/min.

Composition of mobile phase: A-methanol; B-5 mM ammonium formate aqueous solution; ratio A-30%; B-70%.

Injection volume: 3 µl.

UV/vis detector wavelength setting: 265 nm.

The reactant HMF and the main products HMFCA, FFCA and FDCA were well detected under this analytical condition. The conversion of HMF was analyzed and calculated using the standard curve method. Different masses of HMF were weighed with an electronic balance and configured into five standard solutions of different HMF concentrations, which were determined by high performance liquid chromatography under the above operating conditions. The corresponding values of the standard solutions and the integral area were obtained, and linear regression analysis was performed and plotted to obtain a linearly fitted standard curve. The same method was used for the determination and analysis of other products.

The conversion rate of 5-HMF was calculated as follows:

$$Conversion_{HMF}(\%) = \frac{n(HMF\ consumed)}{n(HMF\ initial)} \times 100\%$$

The selectivity and yield of the main product FDCA were calculated as follows:

$$Selectivity(\%) = \frac{mole\ of\ oxidation\ product}{mole\ of\ reacted\ HMF} \times 100\%$$

$$Yield_{FDCA}(\%) = \frac{n(FDCA\ formed)}{n(HMF\ initial)} \times 100\%$$

The Faraday efficiency was calculated as follows:

$$Faradaic\ efficiency(\%) = \frac{n(FDCA\ formed)}{Charge/(6 \times F)} \times 100\%$$

where F was the Faraday constant (96485 C $mol^{-1}$) and n was the mol of reactant calculated from the concentration measured by HPLC.

**Statistical Analysis**

1. Pre-processing of data: XRD, XPS, XAFS, EPR, Raman, BET, ATR-FTIR, HPLC, LSV, CV, OCP, *in-situ* EIS and other electrochemical data were converted into TXT format by the corresponding instruments, and plotted by Origin software without normalization and evaluation of outliers. The data of SEM, TEM, EDX Mapping, Zeta potential, ICP-OES and DFT calculations were in the form of pictures or numbers, which were directly drawn by Word, Powerpoint software without conversion, normalization and evaluation of outliers.

2. Data presentation: The related electrochemical HMF conversion measurement was repeated three times in order to avoid any incidental error. And the related error bars were analyzed by Origin software and shown in the revised supporting information.

3. Sample size for each statistical analysis: The sample size of electrochemical measurement was three.

4. Statistical methods used to assess significant differences with sufficient details: The statistical test was two-sided testing, the α value was 0.05 and related P value was analyzed by Student's two-side t test and showed in the revised supporting information.

5. Software used for statistical analysis: The related software was Origin, Powerpoint, Word, Excel.

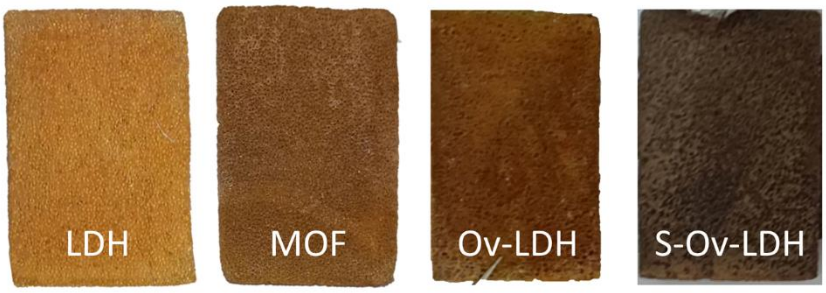


**Figure S1.** The photos of the samples.

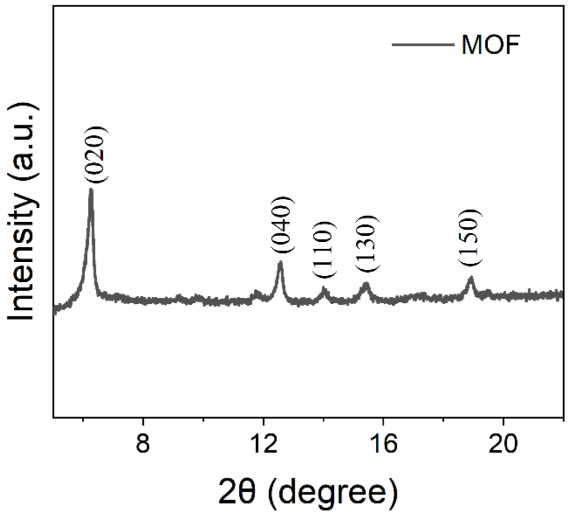


**Figure S2.** XRD pattern of the MOF material.

The correlated XRD result of the MOF material has been given, which is consistent with the result in the previous literature (Adv. Mater. 2020, 32, 2006784), demonstrating the successful transformation of LDH to MOF.

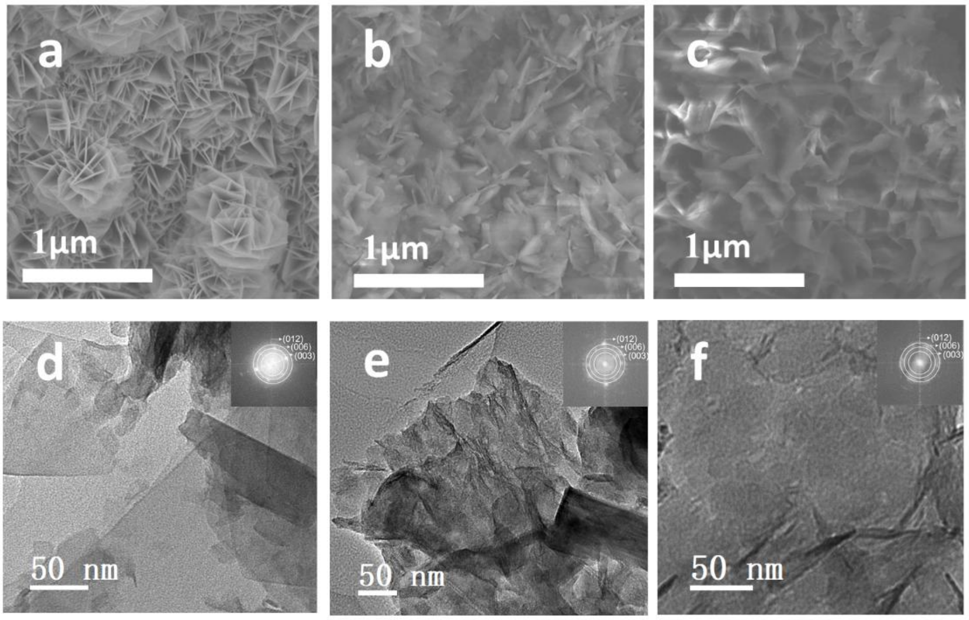


**Figure S3.** SEM, TEM and SAED images of (a, d) LDH, (b, e) Ov-LDH, (c, f) S-Ov-LDH.

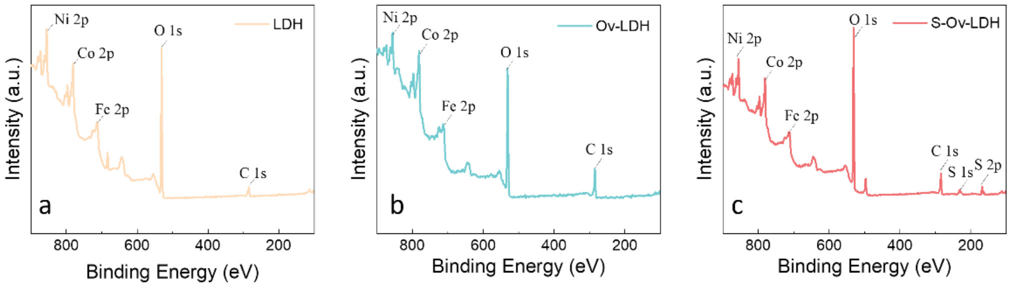


**Figure S4.** XPS survey spectra of (a) LDH, (b) Ov-LDH and (c) S-Ov-LDH.

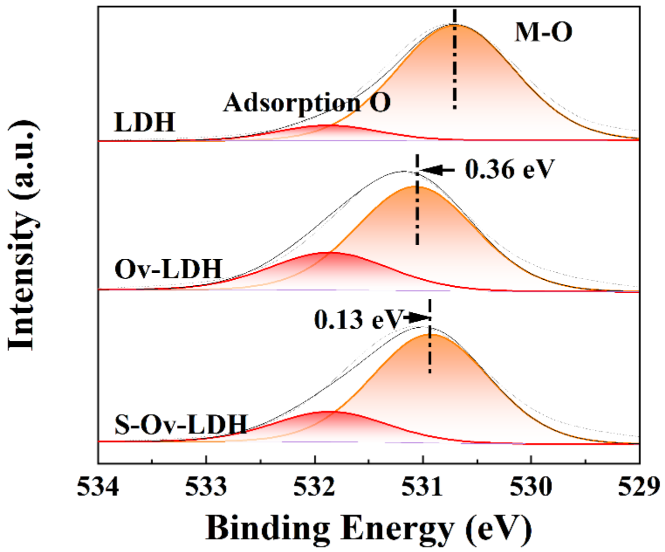


**Figure S5.** O 1s XPS spectrum of LDH, Ov-LDH and S-Ov-LDH.

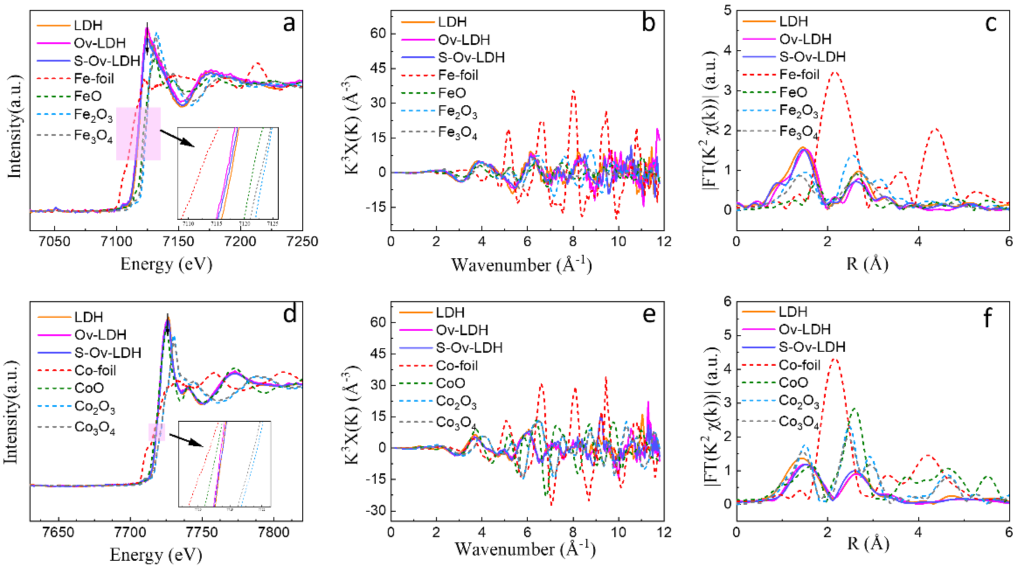

**Figure S6.** (a) XANES spectra, (b) EXAFS K-space and (c) R-space of the (a) related Co-based materials; (d) XANES spectra, (e) EXAFS K-space and (f) R-space of the (a) related Fe-based materials.

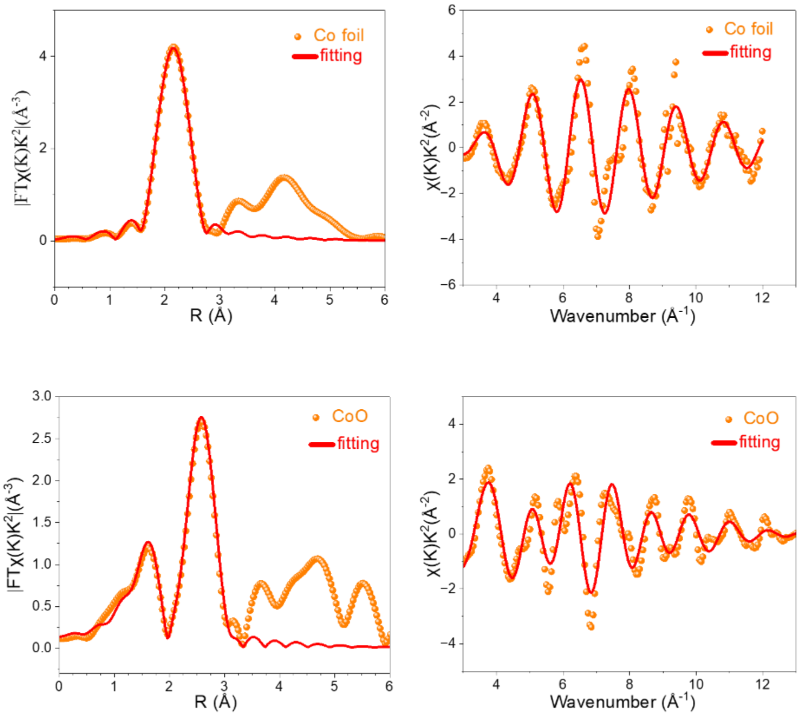


**Figure S7.** EXAFS R-space and K-space fitting curves of Co elements for Co foil and CoO standard samples.

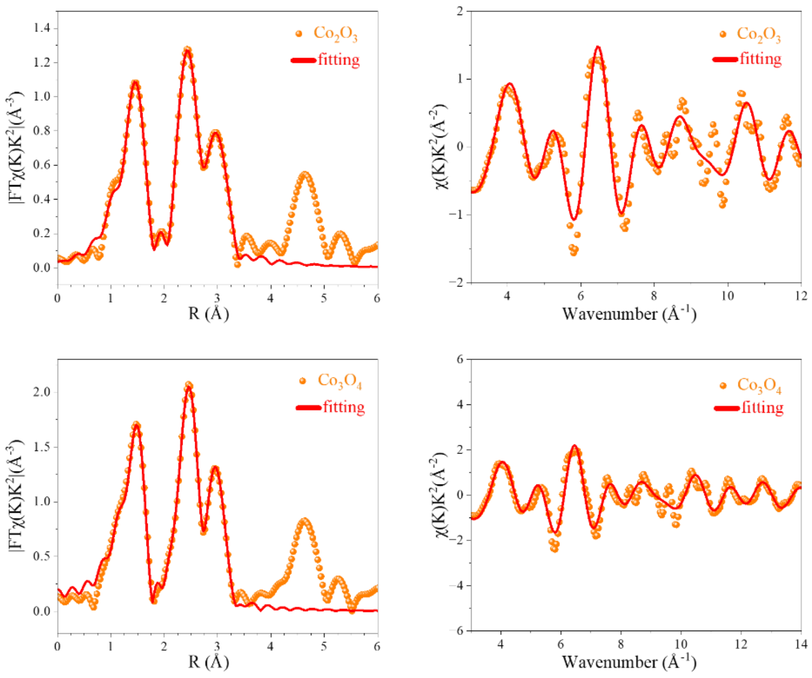

**Figure S8.** EXAFS R-space and K-space fitting curves of Co elements for $Co_2O_3$ and $Co_3O_4$ standard samples.

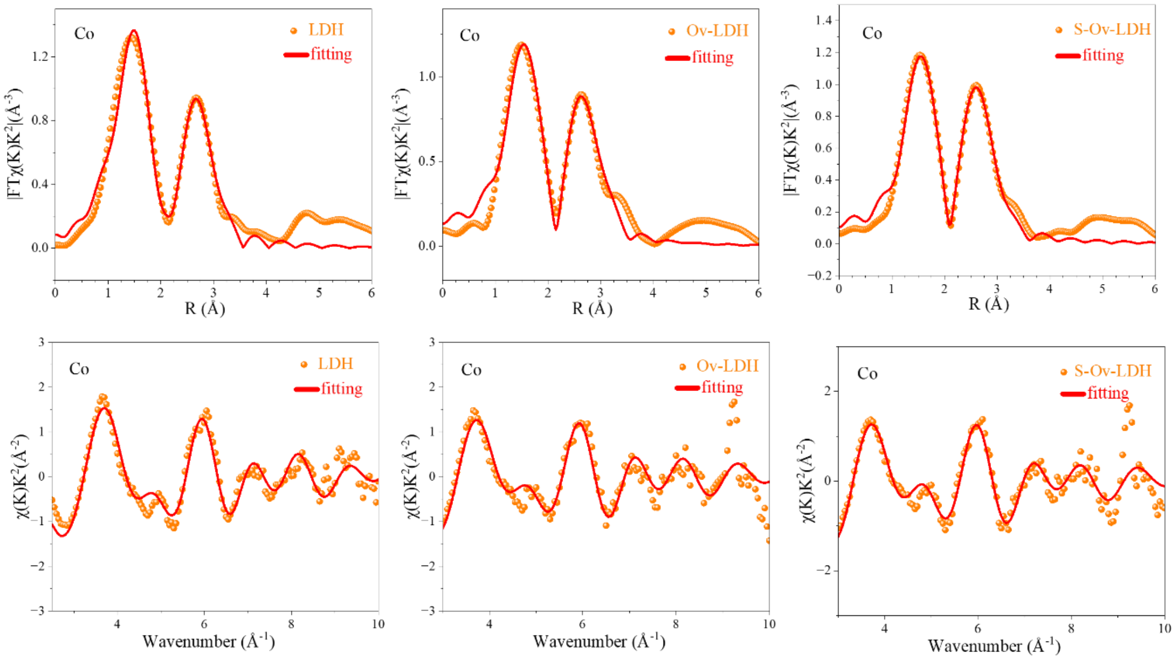


**Figure S9.** EXAFS R-space and K-space fitting curves of Co elements for LDH, Ov-LDH and S-Ov-LDH samples.

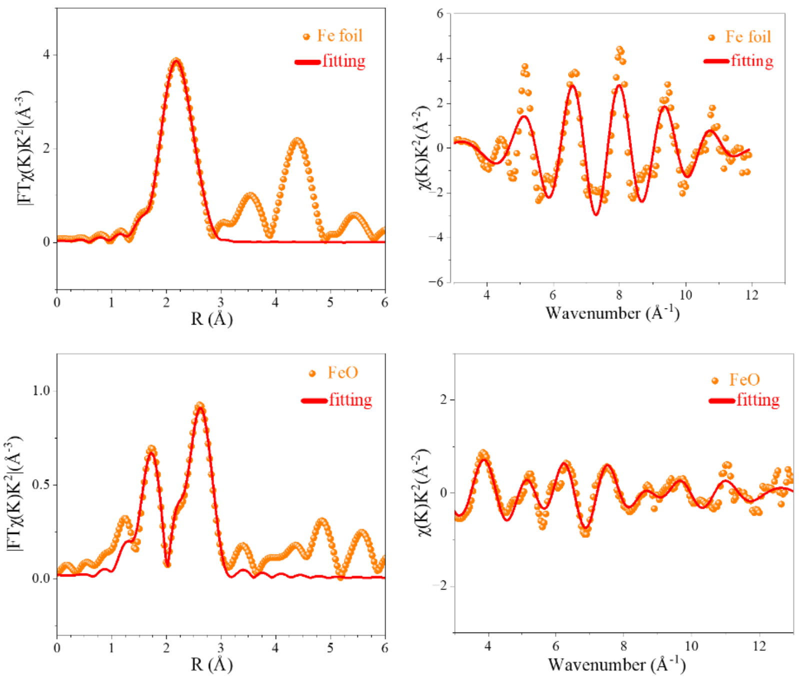

**Figure S10.** EXAFS R-space and K-space fitting curves of Fe elements for Fe foil and FeO standard samples.

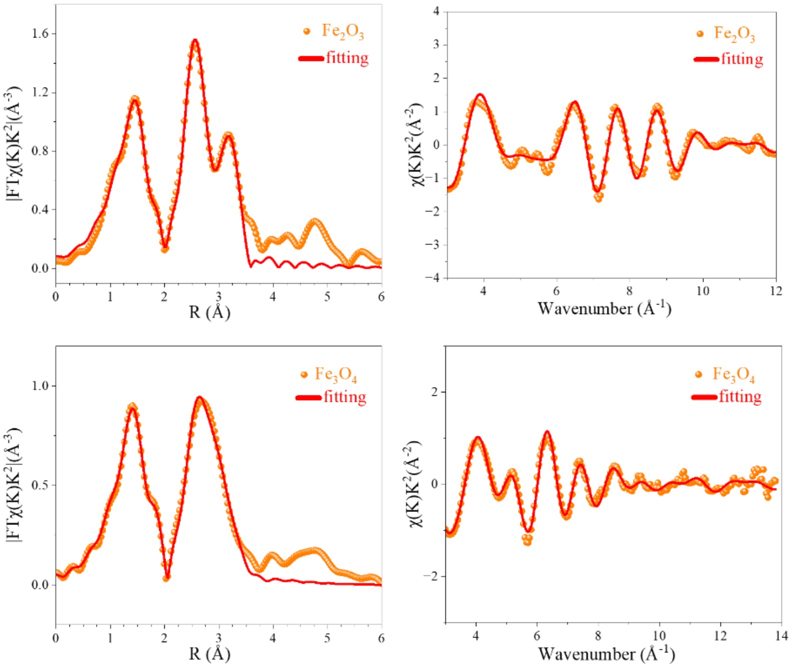


**Figure S11.** EXAFS R-space and K-space fitting curves of Fe elements for $Fe_2O_3$ and $Fe_3O_4$ standard samples.

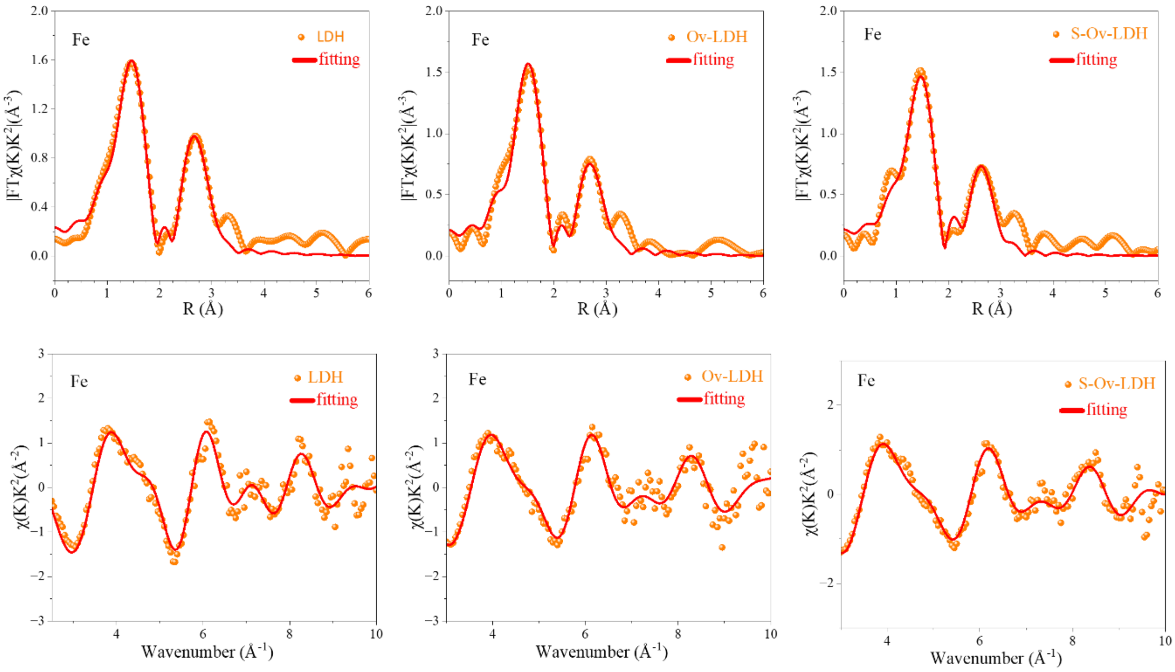

**Figure S12.** EXAFS R-space and K-space fitting curves of Fe elements for LDH, Ov-LDH and S-Ov-LDH samples.

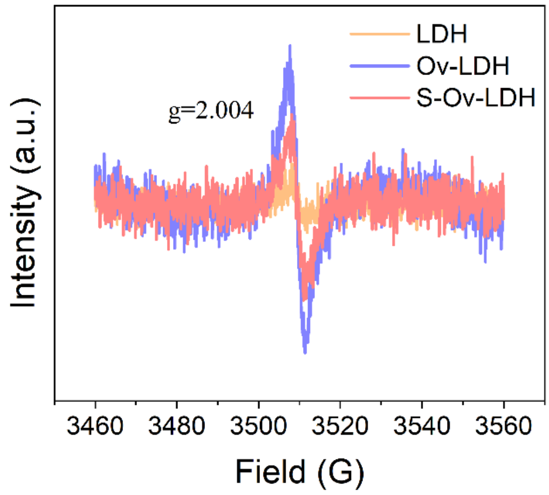


**Figure S13.** Original EPR spectrum of the LDH, Ov-LDH and S-Ov-LDH samples.

The defect concentration values of LDH, Ov-LDH and S-Ov-LDH materials are 1.08 $*10^{11}$ spins/mm$^3$, 2.84 $*10^{11}$ spins/mm$^3$, 1.23 $*10^{11}$ spins/mm$^3$ respectively.

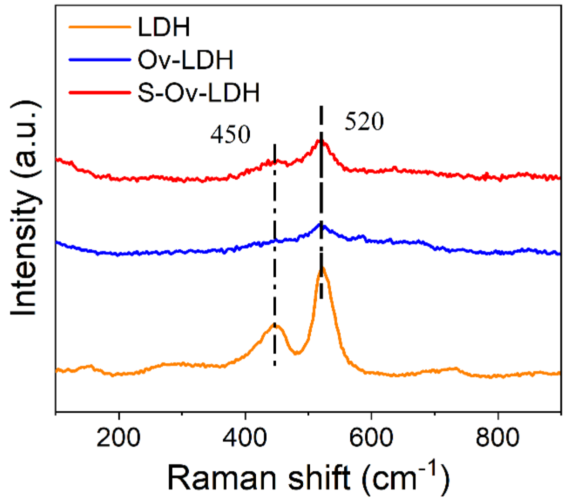


**Figure S14.** Raman spectra of the three samples.

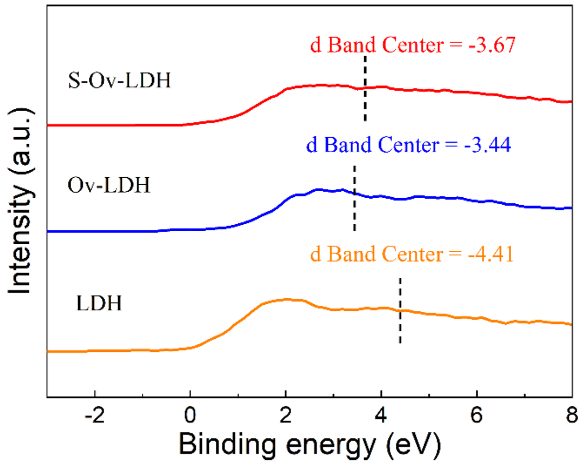


**Figure S15.** d-band center of LDH, Ov-LDH and S-Ov-LDH calculated from XPS valence band spectra.

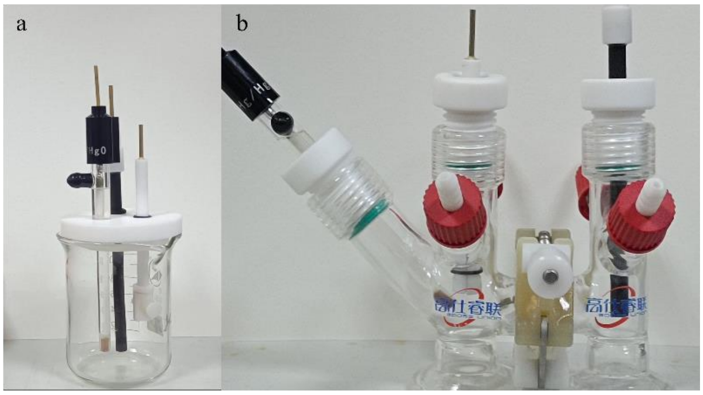


**Figure S16.** The related images of the (a) undivided cell and (b) H-type electrolytic cell.

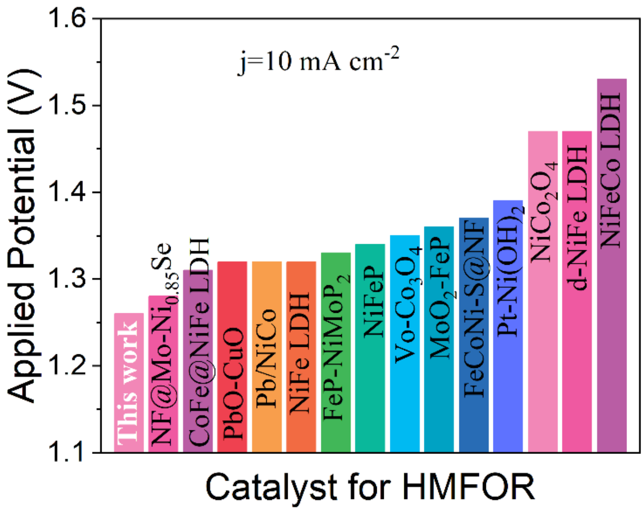


**Figure S17.** The comparison of required applied potential of various catalysts for HMFOR at 10 mA $cm^{-2}$.

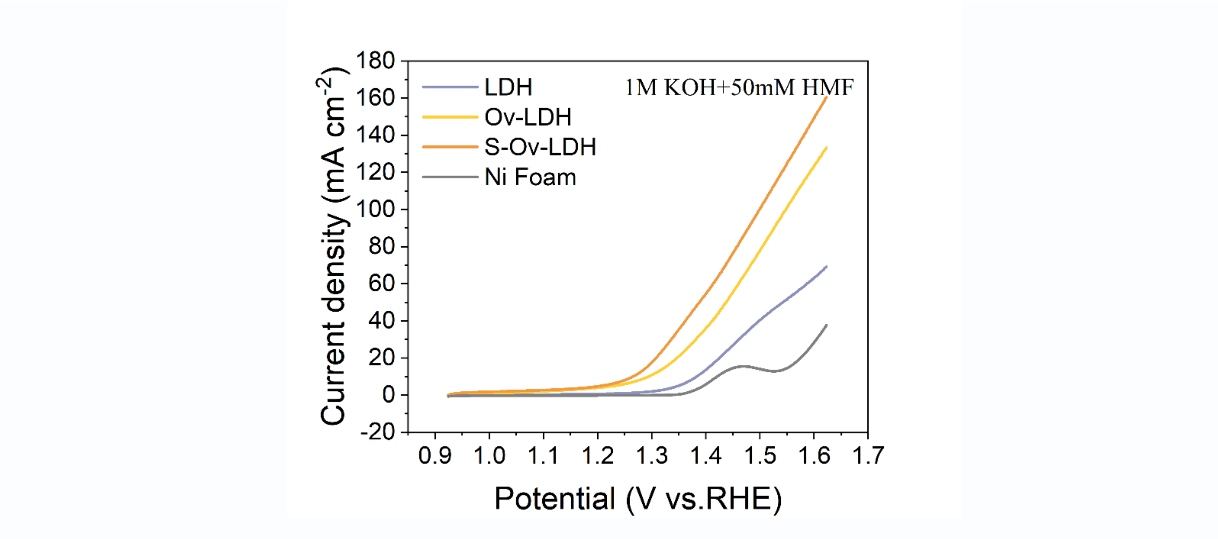


**Figure S18.** LSV polarization curves of the pure Ni foam, LDH, Ov-LDH and S-Ov-LDH samples for HMFOR.

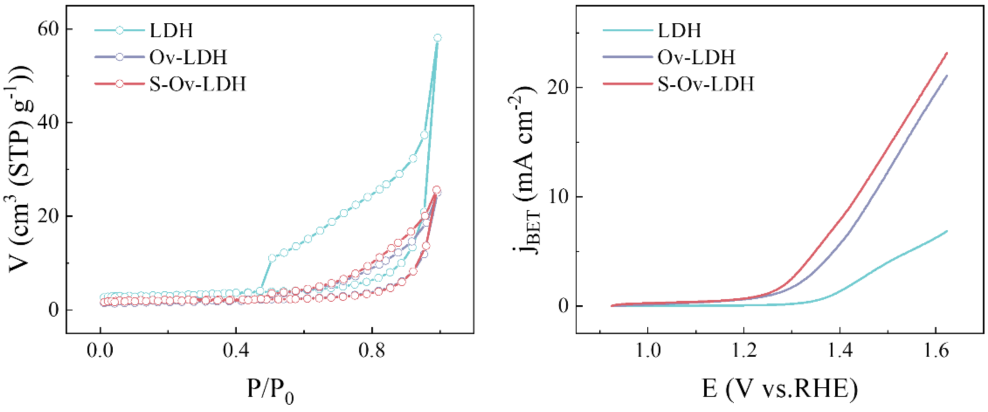


**Figure S19.** The BET results of the three samples (10.10 $cm^3$ $g^{-1}$ for LDH, 6.32 $cm^3$ $g^{-1}$ for Ov-LDH and 6.93 $cm^3$ $g^{-1}$ for S-Ov-LDH), and the normalized LSV curves of the three samples.

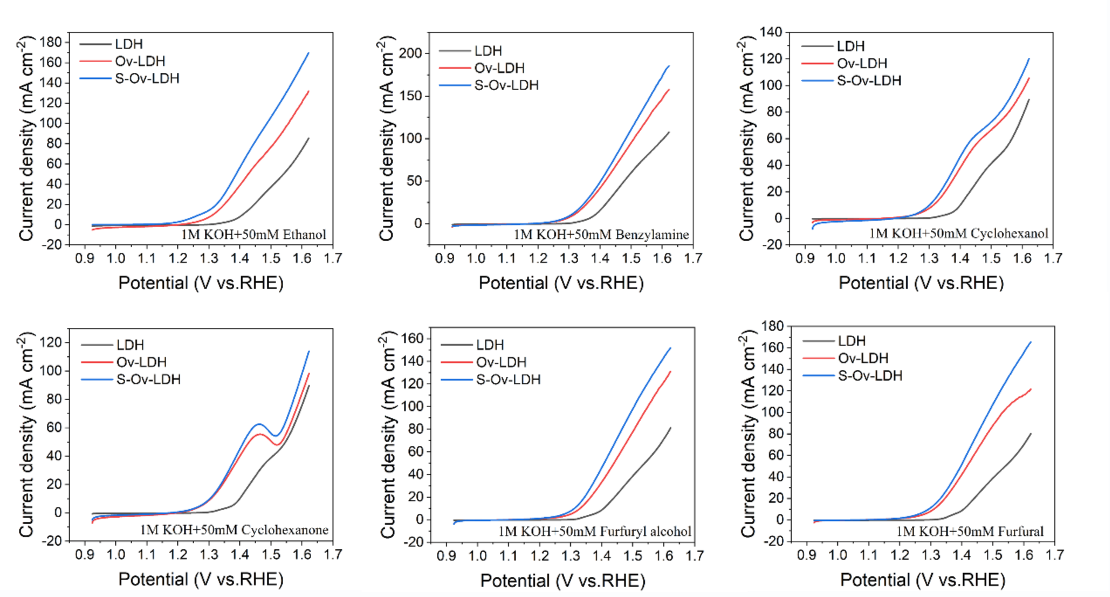


**Figure S20.** LSV curves of the LDH, Ov-LDH and S-Ov-LDH for ethanol, cyclohexanol, cyclohexanone, furfuryl alcohol, furfural, benzylamine oxidation reaction.

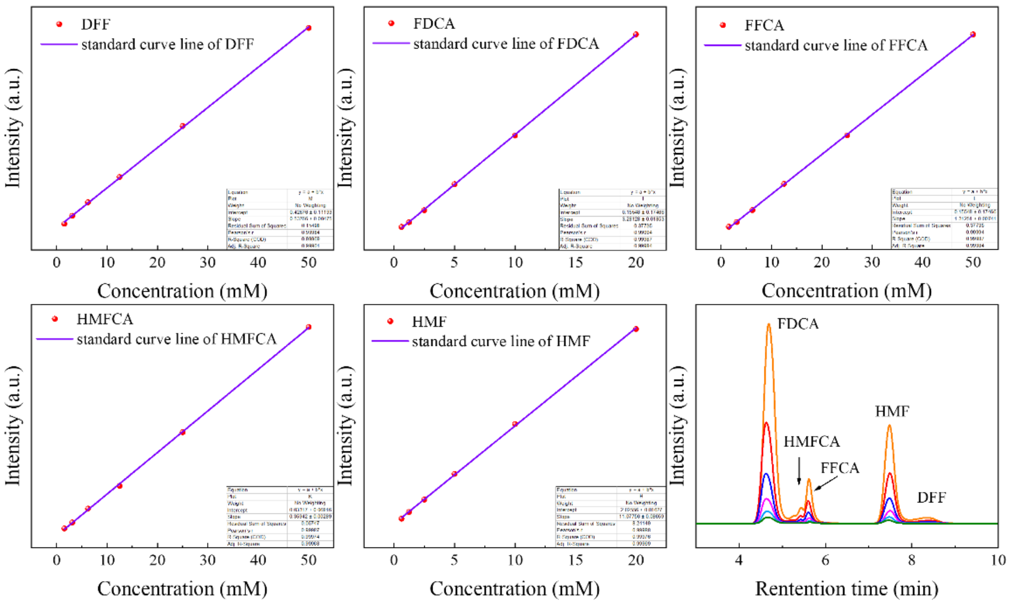


**Figure S21.** The HPLC standard curves of HMF, HMFCA, FFCA, DFF and FDCA, and the related HPLC spectrum.

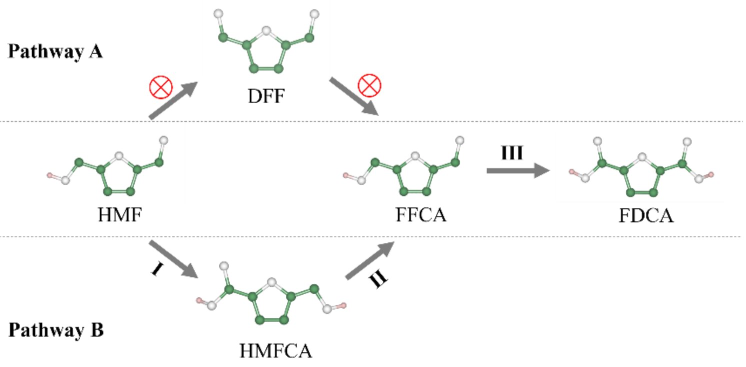


**Figure S22.** The reaction path of the sample in the process of HMFOR.

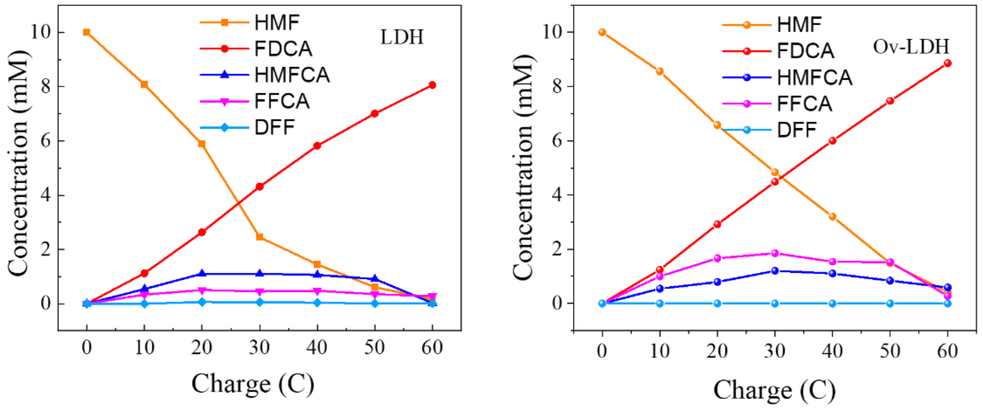


**Figure S23.** The concentration of substrates, intermediates, and products during the HMFOR for LDH and Ov-LDH.

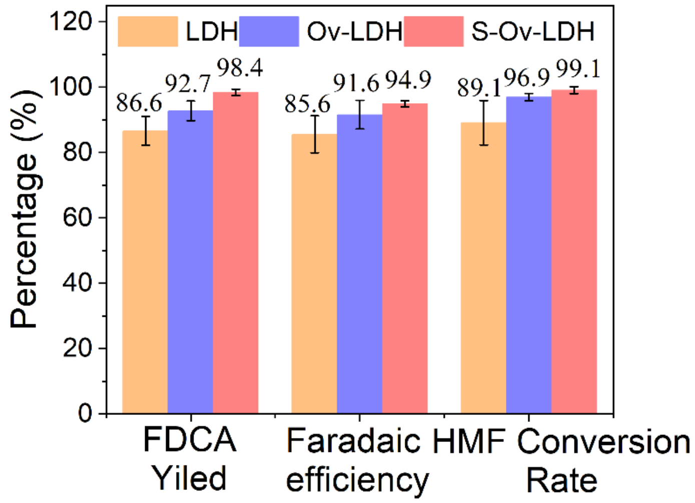


**Figure S24.** The comparison of FDCA yield, Faraday efficiency and HMF conversion rate of the three samples (with error bar, sample size n is 3, P < 0.0001).

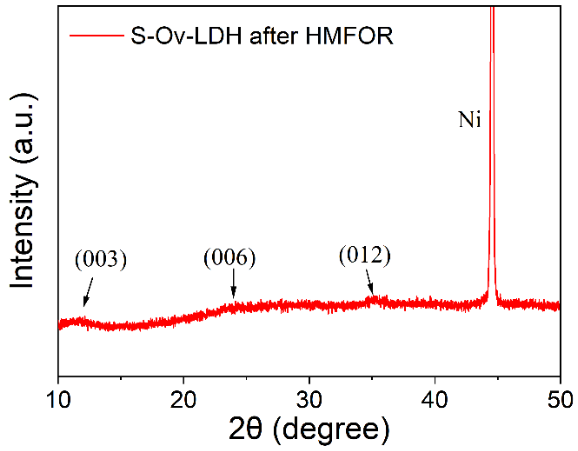


**Figure S25.** XRD patterns of S-Ov-LDH after HMFOR.

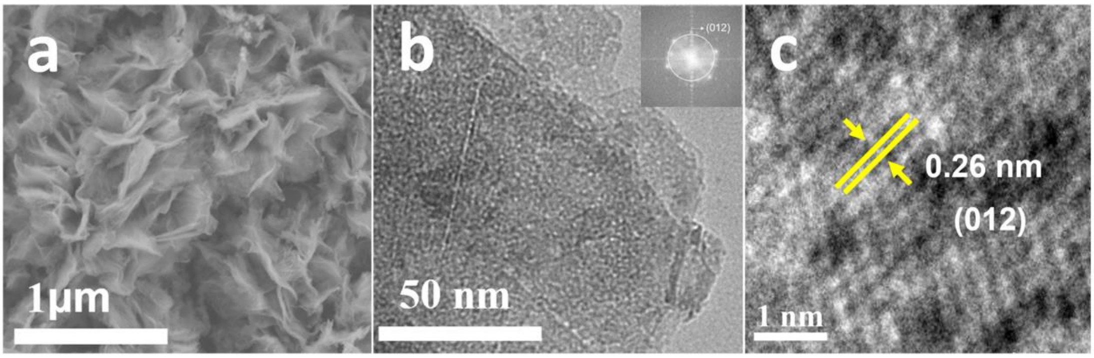


**Figure S26.** (a) SEM (b) TEM and (c) HRTEM of S-Ov-LDH after HMFOR.

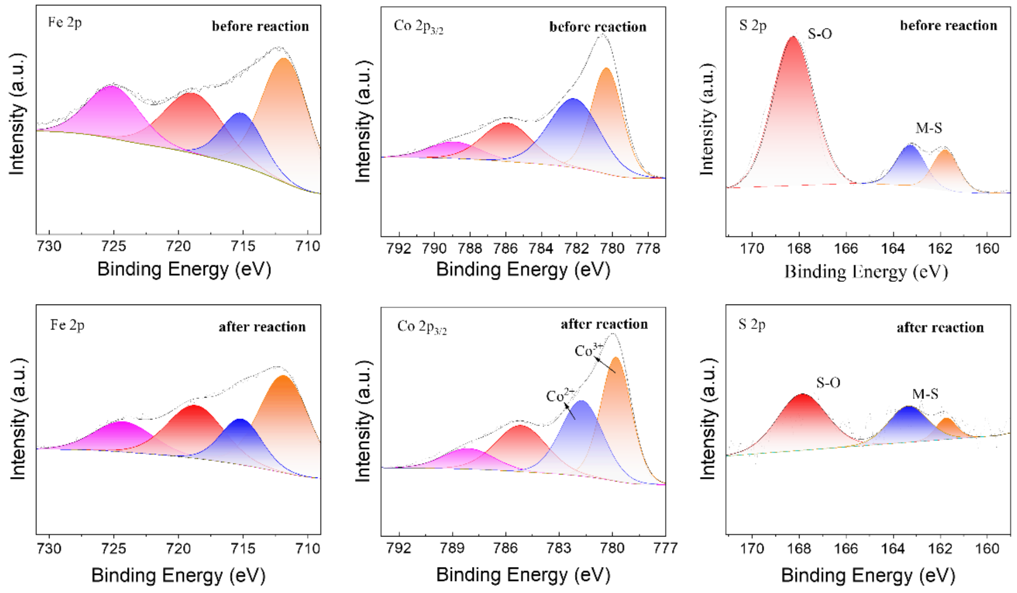


**Figure S27.** Fe 2p, Co 2p and S 2p XPS spectra of the S-Ov-LDH sample after HMFOR.

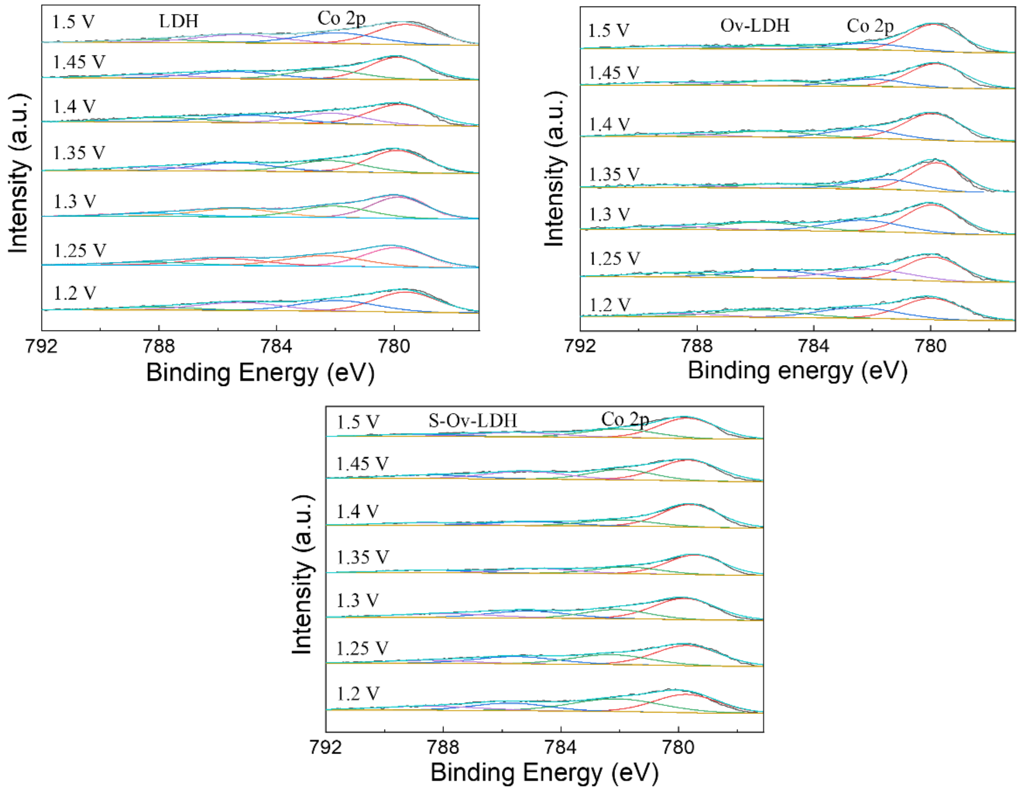


**Figure S28.** Quasi-operando Co 2p XPS spectra of all the three samples with potential from 1.2 V to 1.5 V, an interval of 0.05 V.

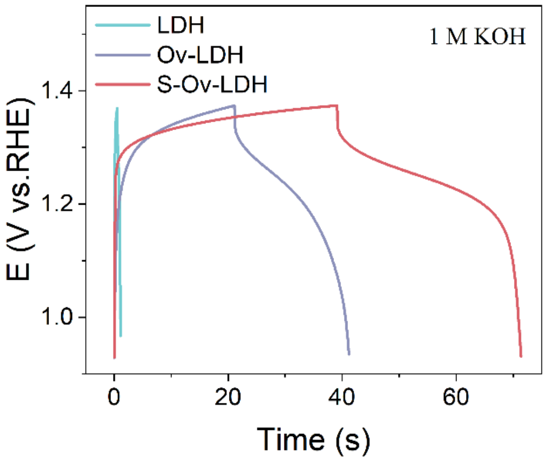


**Figure S29.** The charge and discharge curves of the three samples in the low potential range.

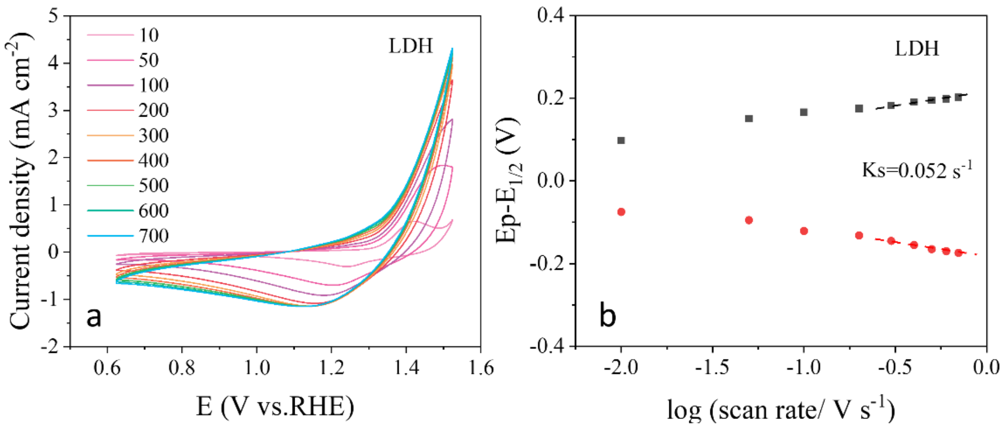


**Figure S30.** Analysis of LDH in Laviron equation. (a) CV curves of LDH with scan rates from 10, 50, 100, 200, 300, 400, 500, 600 to 700 mV $s^{-1}$, 1 M KOH; (b) The plots of the redox peak current densities versus the square root of scan rates.

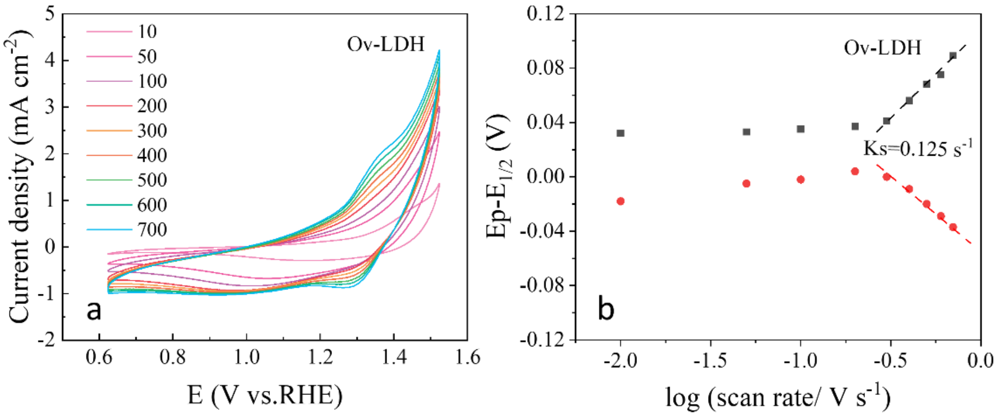


**Figure S31.** Analysis of Ov-LDH in Laviron equation. (a) CV curves of Ov-LDH with scan rates from 10, 50, 100, 200, 300, 400, 500, 600 to 700 mV $s^{-1}$, 1 M KOH; (b) The plots of the redox peak current densities versus the square root of scan rates.

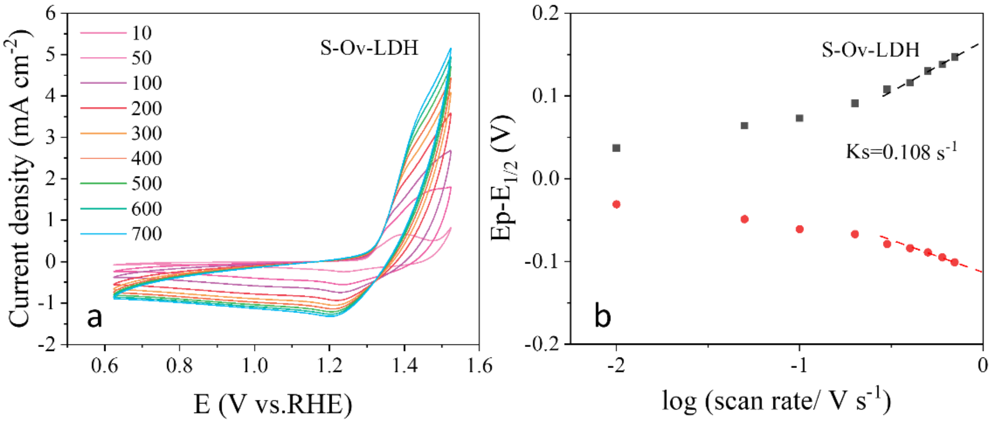


**Figure S32.** Analysis of S-Ov-LDH in Laviron equation. (a) CV curves of S-Ov-LDH with scan rates from 10, 50, 100, 200, 300, 400, 500, 600 to 700 mV $s^{-1}$, 1 M KOH; (b) The plots of the redox peak current densities versus the square root of scan rates.

Ks is calculated by Laviron equation.

Laviron equation is as follows:

$E_c = E_{1/2} - (RT/anF) * \ln(anF/RTKs) - (RT/anF) * \ln(v)$

where $E_c$ is the reduction potential of metal redox, $E_{1/2}$ is the formal potential of metal redox, R is the universal gas constant, T is the temperature in kelvin, n is the number of electrons transferred, a is the transfer coefficient, Ks is the rate constant of metal redox, and v is the scan rate in the CV measurements.

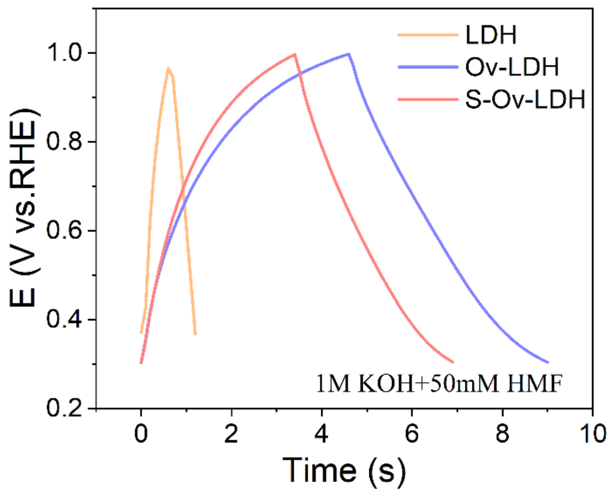


**Figure S33.** The charge and discharge curves of the three samples in the low potential range.

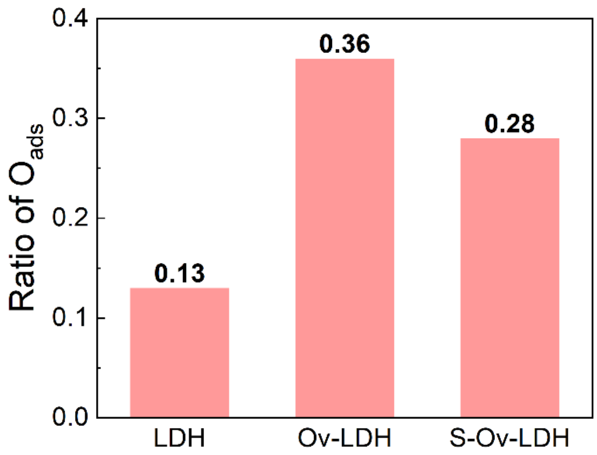


**Figure S34.** The adsorption oxygen ratio of the three samples calculated from O1s XPS spectra.

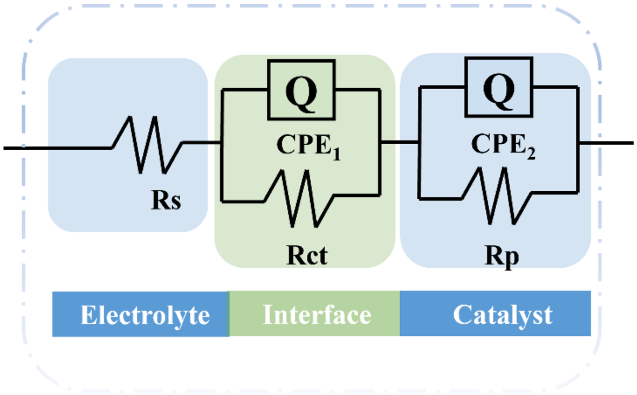


**Figure S35.** The related fitted circuit diagram. **Note:** Rs stands for the solution resistance, CPE1 represents double-layer capacitance, Rct has contact with the interfacial charge transfer reaction, CPE2 and Rp are related to the dielectric properties and the resistance of the electrode inner film.

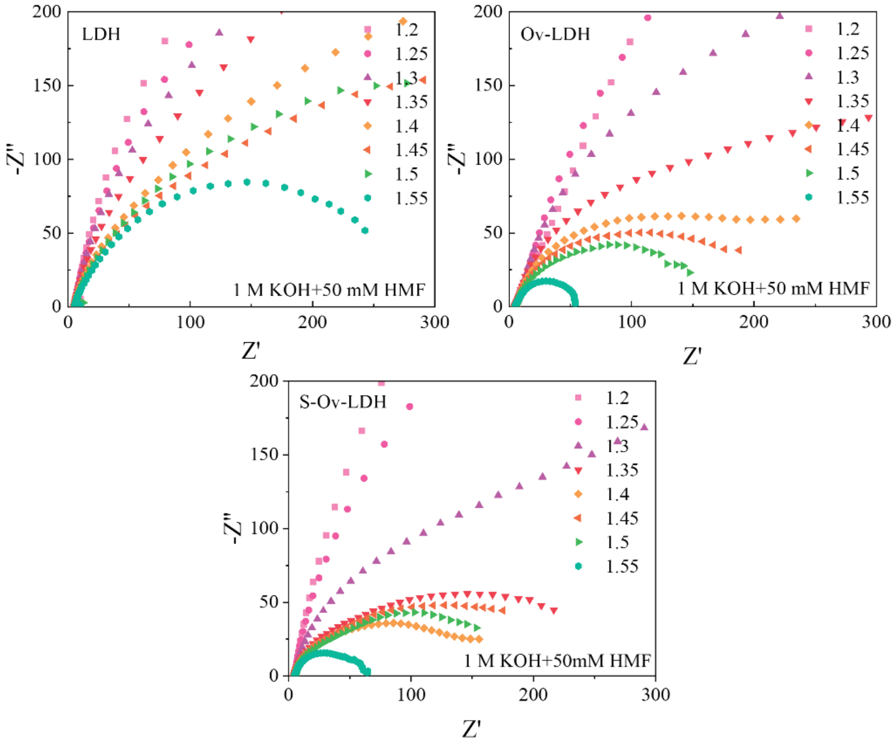


**Figure S36.** Nyquist plots of LDH, Ov-LDH and S-Ov-LDH in 1 M KOH with 50 mM HMF.

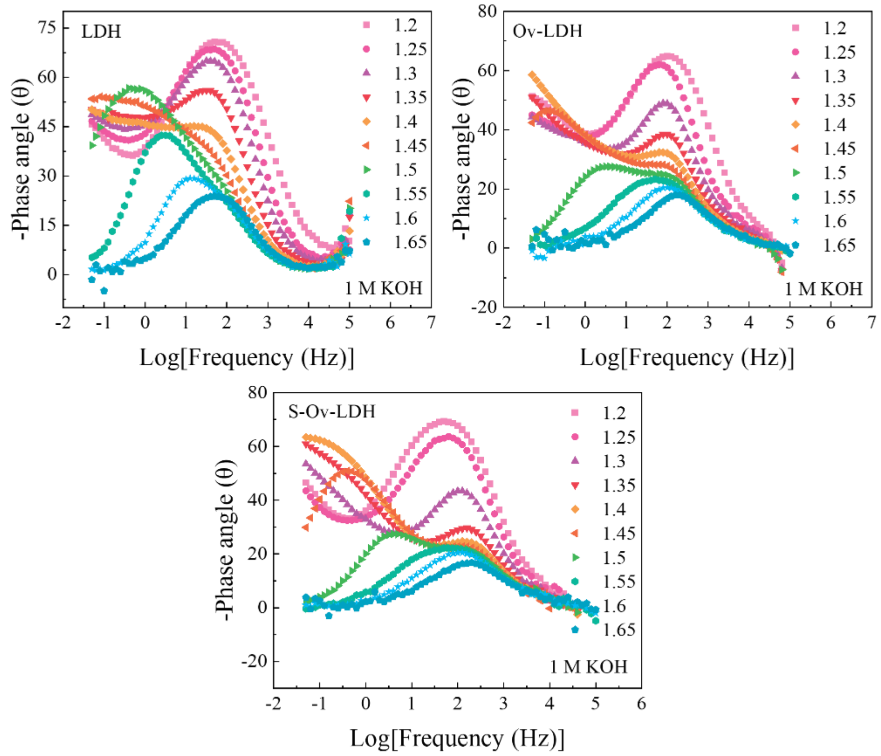


**Figure S37.** Bode plots of LDH, Ov-LDH and S-Ov-LDH in 1 M KOH.

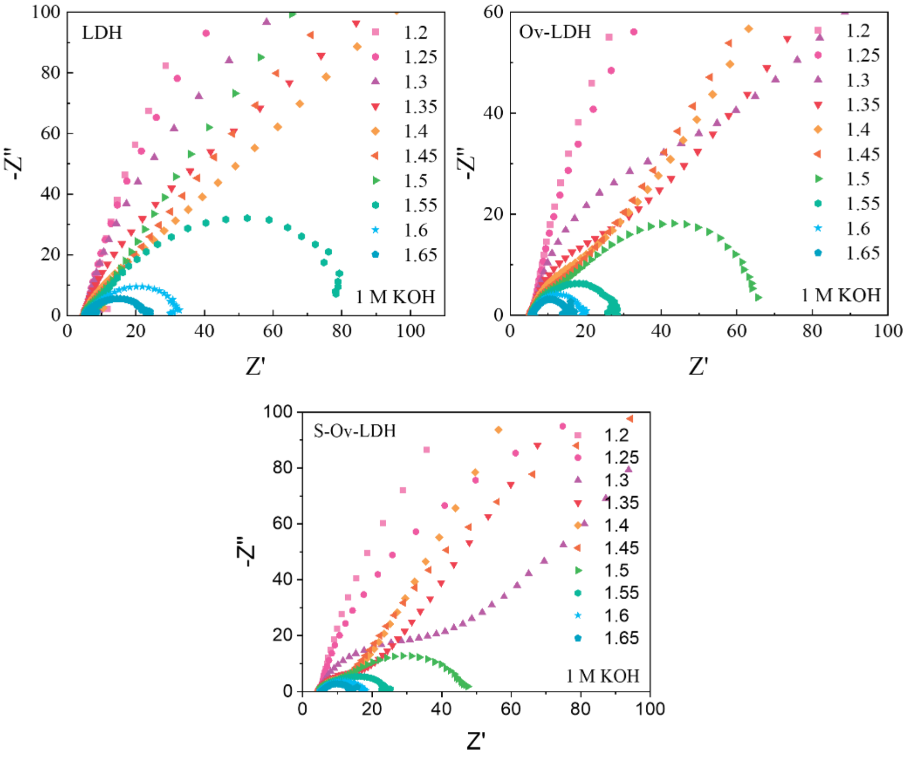


**Figure S38.** Nyquist plots of LDH, Ov-LDH and S-Ov-LDH in 1 M KOH.

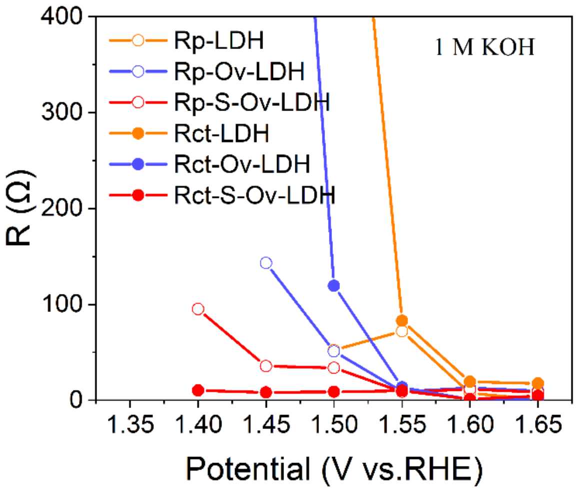


**Figure S39.** Rct and Rp of LDH, Ov-LDH and S-Ov-LDH in 1M KOH.

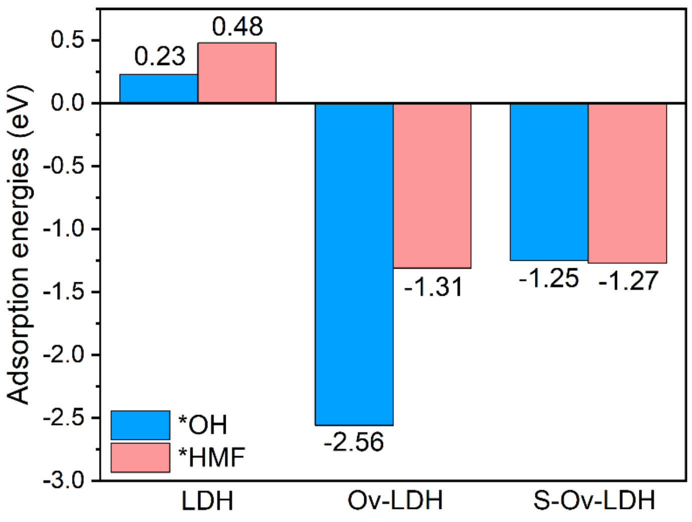


**Figure S40.** HMF and OH adsorption free energy of LDH, Ov-LDH and S-Ov-LDH.

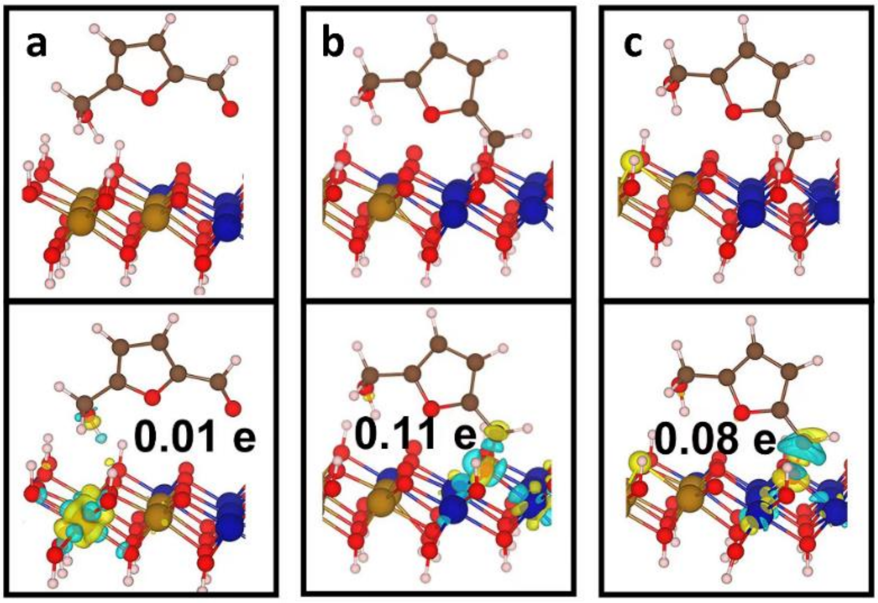


**Figure S41.** Bader charge diagram of HMF adsorbed by (a) LDH, (b) Ov-LDH and (c) S-Ov-LDH.

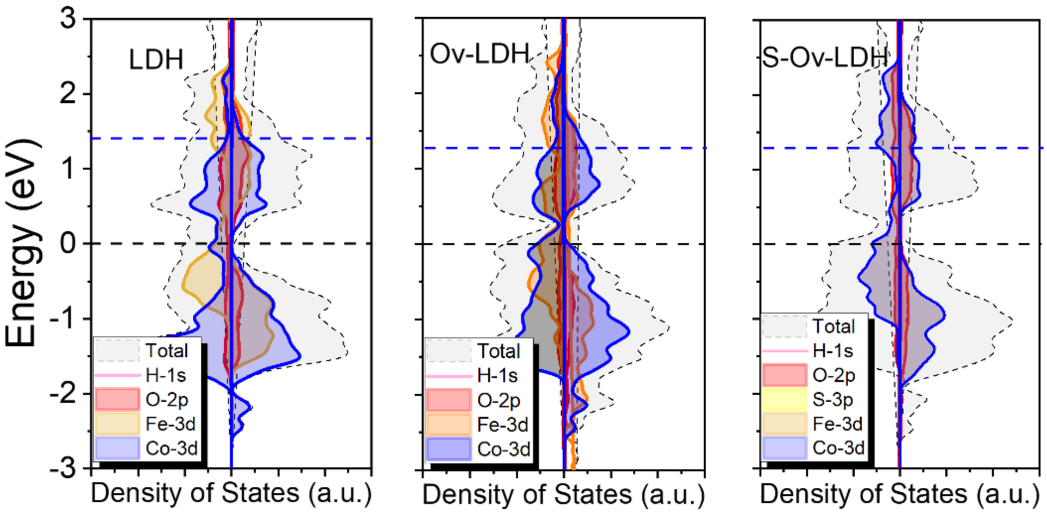


**Figure S42.** The total density of states (TDOS) of LDH, Ov-LDH and S-Ov-LDH.

**Table S1.** ICP-OES result of S-Ov-LDH sample.

| **Atom%** | **Fe** | **Co** | **S** |
|---|---|---|---|
| S-Ov-LDH | 18.9 | 22.2 | 1.8 |

**Table S2.** Structure parameters extracted from the EXAFS fitting of Co K-edge.

| **Sample** | **Shell** | ***CN***[a] | ***R*(Å)**[b] | **$\sigma^2$(Å$^2$)**[c] | **$\Delta E_0$(eV)**[d] | ***R* factor** |
|---|---|---|---|---|---|---|
| Co foil | Co-Co | 12.0* | 2.49±0.01 | 0.0061 | 6.6 | 0.0011 |
| CoO | Co–O | 6.0* | 2.12±0.01 | 0.0070 | 0.3 | 0.0034 |
| | Co-Co | 18.0* | 3.01±0.01 | 0.0101 | -2.7 | |
| $Co_2O_3$ | Co-O | 2.6±0.1 | 1.91±0.01 | 0.0011 | 2.6 | 0.0013 |
| | Co-Co1 | 2.6±0.2 | 2.85±0.01 | 0.0032 | -8.7 | |
| | Co-Co2 | 4.9±0.4 | 3.35±0.01 | 0.0052 | | |
| $Co_3O_4$ | Co-O | 4.1±0.3 | 1.92±0.01 | 0.0019 | -5.6 | 0.0047 |
| | Co-Co1 | 3.8±0.5 | 2.85±0.01 | 0.0032 | -8.2 | |
| | Co-Co2 | 9.2±1.4 | 3.35±0.01 | 0.0071 | -9.7 | |
| LDH | Co–O | 9.1±0.6 | 2.05±0.01 | 0.0131 | -6.0 | 0.0153 |
| | Co-Co | 10.1±1.3 | 3.17±0.01 | 0.0135 | 1.5 | |
| Ov-LDH | Co–O | 6.9±0.5 | 2.11±0.01 | 0.0124 | 0.4 | 0.0140 |
| | Co-Co | 10.8±1.4 | 3.17±0.01 | 0.0140 | 0.8 | |
| S-Ov-LDH | Co–O | 5.5±0.2 | 2.10±0.01 | 0.0089 | 0.8 | 0.0074 |
| | Co-Co | 11.0±0.8 | 3.15±0.01 | 0.0145 | 2.2 | |
| | Co-S | 4.3±1.1 | 3.42±0.01 | 0.0200 | -1.0 | |

**Table S3.** Structure parameters extracted from the EXAFS fitting of Fe K-edge.

| Sample | Shell | $CN^a$ | $R$(Å)$^b$ | $\sigma^2$(Å$^2$)$^c$ | $\Delta E_0$(eV)$^d$ | $R$ factor |
|---|---|---|---|---|---|---|
| Fe foil | Fe-Fe1 | 8.0* | 2.47±0.01 | 0.0046 | -3.2 | 0.0050 |
| | Fe-Fe2 | 6.0* | 2.85±0.02 | 0.0039 | -3.23 | |
| FeO | Fe–O1 | 3.7±0.5 | 2.17±0.01 | 0.0025 | 12.5 | 0.0055 |
| | Fe-O2 | 5.6±1.1 | 2.36±0.01 | 0.0108 | 1.1 | |
| | Fe-Fe | 6.1±0.7 | 3.03±0.01 | 0.0108 | -1.3 | |
| $Fe_2O_3$ | Fe–O1 | 6.7±0.3 | 1.96±0.01 | 0.0081 | -5.7 | 0.0091 |
| | Fe-O2 | 1.6±0.5 | 2.14±0.01 | 0.0029 | -5.4 | |
| | Fe-Fe1 | 6.8±0.3 | 2.99±0.01 | 0.0064 | -0.2 | |
| | Fe-Fe2 | 3.9±0.3 | 3.45±0.01 | 0.0037 | 13.6 | |
| $Fe_3O_4$ | Fe–O1 | 3.9±0.6 | 1.91±0.01 | 0.0045 | -0.5 | 0.0100 |
| | Fe-O2 | 3.0±0.7 | 2.07±0.01 | 0.0038 | -6.0 | |
| | Fe-Fe1 | 7.6±0.5 | 3.02±0.01 | 0.0144 | -0.6 | |
| | Fe-Fe2 | 4.6±0.5 | 3.48±0.01 | 0.0085 | -1.6 | |
| LDH | Fe-O | 6.3±0.7 | 1.99±0.01 | 0.0060 | -4.7 | 0.0100 |
| | Fe-Fe | 7.9±2.4 | 3.15±0.01 | 0.0012 | -0.5 | |
| Ov-LDH | Fe-O | 4.8±0.2 | 2.01±0.01 | 0.0025 | -1.2 | 0.0114 |
| | Fe-Fe | 5.8±0.8 | 3.14±0.01 | 0.0116 | 2.0 | |
| S-Ov-LDH | Fe-O | 5.1±0.2 | 1.98±0.01 | 0.0044 | -4.0 | 0.0119 |
| | Fe-Fe | 2.5±0.3 | 3.10±0.01 | 0.0036 | -2.6 | |
| | Fe-S | 1.1±0.3 | 2.78±0.01 | 0.0055 | 14.1 | |

$^a CN$, coordination number; $^b R$, distance between absorber and backscatter atoms; $^c \sigma^2$, Debye-Waller factor to account for both thermal and structural disorders; $^d \Delta E_0$, inner potential correction; $R$ factor indicates the goodness of the fit. $S_0^2$ was fixed to 0.762. A reasonable range of EXAFS fitting parameters: $0.700 < S_0^2 < 1.000$; $CN > 0$; $\sigma^2 > 0$ Å$^2$; $|\Delta E_0| < 15$ eV; $R$ factor < 0.02.

**Table S4.** The comparison of the electrocatalytic performance of S-Ov-LDH and recently reported electrocatalysts for the oxidation of HMF.

| **Catalyst** | **Electrolyte/HMF concentration[ $\times 10^{-3}$ M]** | **E[V vs.RHE]@Current density(mA $cm^{-2}$)** | **FE[%]@E[V vs.RHE]** | **References** |
|---|---|---|---|---|
| LDH | 1 M KOH/50 | 1.38 @ 10 | 77.8 @ 1.42 | This work |
| S-Ov-LDH | 1 M KOH/50 | 1.26 @ 10 | 95 @ 1.42 | This work |
| Pd/NiCo | 1 M KOH/50 | 1.32 @ 10 | 97 @ 1.45 | *Angew. Chem. Int. Ed.* 2023, 135, 11696. |
| $Pd/NiCo_2O_4$ | 1M KOH/50 | 1.2 @ 10 | 99.6 @ 1.5 | *Nano-Micro Lett.* 2024, *16*, 275 |
| $V_{mo}$-$NiO_xH_y$ | 1M KOH/10 | 1.35 @ 10 | 93 @ 1.4 | *Appl. Catal. B: Environ and Energy.* 2024, *347*, 123785 |
| NiFe LDH | 1 M KOH/10 | 1.24 @ 20 | 90 @ 1.42 | *Adv. Mater.* 2023, 35, 2305573. |
| $NiCo_2O_4$ | 1 M KOH/5 | 1.47 @ 10 | 87.5 @ 1.5 | *Appl. Catal. B-Environ.* 2019, 242, 85. |
| CoNiP | 1 M KOH/10 | 1.46 @ 20 | 87.2 @ 1.5 | *Appl. Catal. B-Environ.* 2022, 312, 121400. |
| d-NiFe LDH | 1 M KOH/10 | 1.47 @ 10 | 84.5 @ 1.5 | *ACS. Sustain. Chem. Eng.* 2021, 10, 645. |
| CoOOH@ $CeO_2$ | 1M KOH/50 | 1.29 @ 50 | 97 @ 1.4 | *J. Energy Chem.* 2024, *98*, 721-732. |
| $Pt$-$Ni(OH)_2$ | 1 M KOH/50 | 1.39 @ 10 | 98.7 @ 1.45 | *Angew. Chem. Int. Ed.* 2021, 60, 22908. |
| $MoO_2$-FeP | 1 M KOH/10 | 1.36 @ 10 | 97.8 @ 1.42 | *Adv. Mater.* 2020, 32, 2000455. |

| | | | | |
|---|---|---|---|---|
| $NiS_x$-$Ni_2P$ | 1 M KOH/10 | 1.35 @ 20 | 95.1 @ 1.46 | *Green Chem.* 2022, 24, 877. |
| FeP-$NiMoP_2$ | 1 M KOH/10 | 1.33 @ 10 | 99 @ 1.4 | *Nat Commun.* 2022, 13, 3125. |
| Ni-Cu/NF | 1M KOH/50 | 1.32 @ 10 | 95 @ 1.5 | *Angew Chem Int Ed.* 2023, *62*, 37. |
| $Mn_{0.2}$NiS/GF | 1M KOH/100 | 1.25 @ 10 | 94.2 @ 1.4 | *Adv Funct Mater.* 2023, *33*, 24. |
| PdO-CuO | 1 M KOH/50 | 1.32 @ 10 | 93.7 @ 1.4 | *Adv. Mater.* 2022, 34, 2204089. |
| CoP-CoOOH | 1 M KOH/150 | 1.48 @ 50 | 96.3 @ 1.42 | *Appl. Catal. B-Environ.* 2022, 315, 121588. |
| NiFe LDH | 1 M KOH/10 | 1.32 @ 20 | 99.4 @ 1.23 | *ACS Catal.* 2018, 8, 5533. |
| NiFeCo LDH | 1 M KOH/5 | 1.53 @ 10 | 90 @ 1.54 | *ACS Catal.* 2020, 10, 5179. |
| NiCoMn LDH | 1 M NaOH/1 | 1.6 @ 50 | - | *Green Chem.* 2021, 23, 4034. |
| CoFe@NiFe LDH | 1 M KOH/10 | 1.31 @ 10 | 75.7 @ 1.34 | *Adv. Funct. Mater.* 2021, 31, 2102886. |
| $Cu_xS$@NiCo LDHs | 1 M KOH/10 | 1.34 @ 20 | 90 @ 1.32 | *J. Mater. Chem. A*.2020, 8, 1138. |
| Vo-$Co_3O_4$ | 1 M KOH/10 | 1.35 @ 10 | 88.1 @ 1.47 | *Adv. Mater.* 2021, 34, 2107185. |
| NiFeP | 1 M KOH/10 | 1.34 @ 10 | 1.44 @ 94.6 | *Appl. Catal. B-Environ.* 2022, 311, 121357. |
| NF@Mo-$Ni_{0.85}$Se | 1 M KOH/10 | 1.28 @ 10 | 1.4 @ 95 | *Chem. Eng. J.* 2021, 422, 130125. |
| FeCoNi-S@NF | 1 M KOH/50 | 1.37 @ 10 | 1.45 @ 95 | *Chem. Eng. J.* 2024, 481, 148429. |

**Table S5.** The related EIS fitting parameters of LDH sample for OER.

| Potential | Rs | Rct | CPE0 | N0 | Rp | CPE1 | N1 |
|---|---|---|---|---|---|---|---|
| **1.2** | | | | | | | |
| **1.25** | | | | | | | |
| **1.3** | | | | | | | |

| | | | | | | | |
|---|---|---|---|---|---|---|---|
| **1.35** | | | | | | | |
| **1.4** | | | | | | | |
| **1.45** | | | | | | | |
| **1.5** | 4.87 | 833.5 | 0.004170 | 0.7432 | 52.29 | 0.003719 | 0.7684 |
| **1.55** | 4.833 | 83 | 0.003598 | 0.8737 | 71.95 | 0.004539 | 0.6711 |
| **1.6** | 5.13 | 19.29 | 0.002643 | 0.9077 | 7.444 | 0.002622 | 0.7183 |
| **1.65** | 5.292 | 17.53 | 0.002402 | 0.7069 | 0.9662 | 0.001307 | 0.9662 |

**Table S6.** The related EIS fitting parameters of Ov-LDH sample for OER.

| Potential | Rs | Rct | CPE0 | N0 | Rp | CPE1 | N1 |
|---|---|---|---|---|---|---|---|
| **1.2** | | | | | | | |
| **1.25** | | | | | | | |
| **1.3** | | | | | | | |
| **1.35** | | | | | | | |
| **1.4** | | | | | | | |
| **1.45** | 5.298 | 1125 | 0.007982 | 0.6377 | 143.1 | 0.002184 | 0.6856 |
| **1.5** | 5.484 | 119.3 | 0.001884 | 0.6828 | 51.02 | 0.005489 | 0.7509 |
| **1.55** | 5.521 | 13.63 | 0.002336 | 0.6555 | 9.02 | 0.006706 | 0.8085 |
| **1.6** | 5.809 | 0.7796 | 0.04196 | 0.9979 | 13.02 | 0.001392 | 0.7088 |
| **1.65** | 5.735 | 0.3765 | 1.172 | 0.9991 | 9.723 | 0.0009817 | 0.7283 |

**Table S7.** The related EIS fitting parameters of S-Ov-LDH sample for OER.

| Potential | Rs | Rct | CPE0 | N0 | Rp | CPE1 | N1 |
|---|---|---|---|---|---|---|---|
| **1.2** | | | | | | | |
| **1.25** | | | | | | | |
| **1.3** | | | | | | | |
| **1.35** | | | | | | | |
| **1.4** | 4.592 | 10.22 | 0.001308 | 0.7174 | 95.04 | 0.006848 | 0.7481 |
| **1.45** | 4.736 | 8.278 | 0.001181 | 0.7522 | 35.55 | 0.000650 | 0.7648 |
| **1.5** | 4.609 | 8.787 | 0.005012 | 0.8082 | 33.83 | 0.001890 | 0.6748 |

| | | | | | | | |
|---|---|---|---|---|---|---|---|
| **1.55** | 5.103 | 9.947 | 0.004212 | 0.6934 | 8.862 | 0.001703 | 0.8703 |
| **1.6** | 5.063 | 1.283 | 0.014080 | 0.7128 | 11.46 | 0.001542 | 0.6981 |
| **1.65** | 5.525 | 4.474 | 0.01301 | 0.7136 | 8.651 | 0.001276 | 0.7128 |

**Table S8.** The related EIS fitting parameters of LDH sample for HMFOR.

| Potential | Rs | Rct | CPE0 | N0 | Rp | CPE1 | N1 |
|---|---|---|---|---|---|---|---|
| **1.2** | | | | | | | |
| **1.25** | | | | | | | |
| **1.3** | | | | | | | |
| **1.35** | | | | | | | |
| **1.4** | 5.156 | 6859 | 0.002432 | 0.76 | 115.6 | 0.001044 | 0.8219 |
| **1.45** | 5.159 | 476.2 | 0.03277 | 0.7781 | 89.19 | 0.03277 | 0.8117 |
| **1.5** | 5.122 | 450.9 | 0.003352 | 0.7785 | 84.97 | 0.002187 | 0.7921 |
| **1.55** | 5.113 | 245 | 0.002567 | 0.7527 | 24.87 | 0.003757 | 0.8195 |

**Table S9.** The related EIS fitting parameters of Ov-LDH sample for HMFOR.

| Potential | Rs | Rct | CPE0 | N0 | Rp | CPE1 | N1 |
|---|---|---|---|---|---|---|---|
| **1.2** | | | | | | | |
| **1.25** | | | | | | | |
| **1.3** | | | | | | | |
| **1.35** | 4.848 | 465.4 | 0.03059 | 0.6459 | 65.16 | 0.001055 | 0.9852 |
| **1.4** | 4.88 | 234.3 | 0.003632 | 0.6444 | 37.87 | 0.001511 | 0.9915 |
| **1.45** | 4.824 | 181.8 | 0.003874 | 0.6538 | 24.2 | 0.002248 | 0.9816 |
| **1.5** | 4.788 | 146.6 | 0.003867 | 0.6592 | 12.59 | 0.003022 | 0.9924 |
| **1.55** | 4.757 | 43.68 | 0.003621 | 0.6790 | 9.32 | 0.003689 | 0.9996 |

**Table S10.** The related EIS fitting parameters of S-Ov-LDH sample for HMFOR.

| Potential | Rs | Rct | CPE0 | N0 | Rp | CPE1 | N1 |
|---|---|---|---|---|---|---|---|
| **1.2** | | | | | | | |
| **1.25** | 5.306 | 2183 | 0.001121 | 0.8475 | 597.2 | 0.0002558 | 0.8882 |
| **1.3** | 4.956 | 1487 | 0.0004298 | 0.8653 | 594.9 | 0.002769 | 0.7719 |

| | | | | | | | |
|---|---|---|---|---|---|---|---|
| **1.35** | 4.790 | 181.8 | 0.0006813 | 0.7354 | 47.53 | 0.003732 | 0.8596 |
| **1.4** | 4.662 | 118.4 | 0.004417 | 0.711 | 35.48 | 0.0009491 | 0.8543 |
| **1.45** | 4.681 | 142.7 | 0.003689 | 0.7206 | 22.37 | 0.001139 | 0.8611 |
| **1.5** | 4.694 | 25.39 | 0.004970 | 0.6748 | 11.91 | 0.001266 | 0.8514 |
| **1.55** | 4.816 | 21.62 | 0.00107 | 0.7932 | 5.52 | 0.001931 | 0.8065 |

**References:**

[1] Kresse, G.; Furthmüller, J. Efficient Iterative Schemes for ab initio Total-energy Calculations Using a Plane-wave Basis Set. *Phys. Rev. B* 1996, *54*, 11169−11186.

[2] Blöchl, P. E. Projector Augmented-Wave Method. *Phys. Rev. B* 1994, *50*, 17953−17979.

[3] Grimme, S.; Ehrlich, S.; Goerigk, L. Effect of the Damping Function in Dispersion Corrected Density Functional Theory. *J. Comput. Chem.* 2011, *32*, 1456−1465.

[4] Grimme, S.; Antony, J.; Ehrlich, S.; Krieg, H. A Consistent and Accurate Ab Initio Parametrization of Density Functional Dispersion Correction (DFT-D) for the 94 Elements H-Pu. *J. Chem. Phys.* 2010, *132*, 154104.

[5] Dudarev, S. L.; Botton, G. A.; Savrasov, S. Y.; Humphreys, C. J.; Sutton, A. P. Electron-energy-loss spectra and the structural stability of nickel oxide: An LSDA+U study. *Phys. Rev. B* 1998, *57*, 1505.

[6] Bi, Yongmin; Cai, Z.; Zhou, D.; Tian, Y.; Zhang, Q.; Zhang, Q.; Kuang, Y.; Li, Y.; Sun, X.; Duan, X. Understanding the incorporating effect of $Co^{2+}/Co^{3+}$ in NiFe-layered double hydroxide for electrocatalytic oxygen evolution reaction. *J. Catal.* 2018, *358*, 100–107.

[7] Li, P.; Wang, M.; Duan, X.; Zheng, L.; Cheng, X.; Zhang, Y.; Kuang, Y.; Li, Y.; Ma, Q.; Feng, Z.; Liu, W.; Sun, X. Boosting oxygen evolution of single-atomic ruthenium through electronic coupling with cobalt-iron layered double hydroxides. *Nat. Commun.* 2019, *10*, 1711.

[8] Hu, Q.; Sun, D.; Wu, Q.; Wang, H.; Wang, L.; Liu, B.; Zhou, A.; He, J. MXene: a new family of promising hydrogen storage medium. *J. Phys. Chem. A* 2013, *117*, 14253.

[9] Tang, W.; Sanville, E.; Henkelman, G. A grid-based Bader analysis algorithm without lattice bias. *J. Phys. Condens. Matter* 2009, *21*, 084204.

[10] Becke, A. D.; Edgecombe, K. E. A simple measure of electron localization in atomic and molecular systems. *J. Chem. Phys.* 1990, *92*, 5397.
